# Cellular immune responses 12 months after fractional or standard dose BNT162b2 booster vaccination in Mongolian adults

**DOI:** 10.3389/fimmu.2026.1779435

**Published:** 2026-04-22

**Authors:** Nadia Mazarakis, Zheng Quan Toh, Tsetsegsaikhan Batmunkh, Jeremy Anderson, Leanne Quah, Yan Yung Ng, Otgonjargal Amraa, Tsogjargal Burentogtokh, Sarantsetseg Jigjidsuren, Bolor Altangerel, Narantuya Namjil, Khaliunaa Mashbaatar, Rachael Carissa, Skyy Luu, Kerryn A. Moore, Eleanor F. G. Neal, Cattram Nguyen, Shuo Li, John Hart, Lien Anh Ha Do, Frances Justice, Kim Mulholland, Claire von Mollendorf, Paul V. Licciardi

**Affiliations:** 1 Infection, Immunity, and Global Health, Murdoch Children’s Research Institute, Melbourne, VIC, Australia; 2 Department of Paediatrics, The University of Melbourne, Parkville, VIC, Australia; 3 National Centre for Communicable Diseases, Ulaanbaatar, Mongolia; 4 General Laboratory of Clinical Pathology, First Central Hospital of Mongolia, Ulaanbaatar, Mongolia; 5 Onoshmed Clinical Laboratory, Ulaanbaatar, Mongolia; 6 London School of Hygiene & Tropical Medicine, London, United Kingdom

**Keywords:** cellular immune response, COVID-19, immunology, longitudinal, vaccine

## Abstract

Despite the role of booster doses in sustaining protection against emerging SARS-CoV-2 variants, global uptake remains low, highlighting the need for dose-sparing strategies that maintain durable immunity. We conducted a randomized controlled trial in Mongolian adults to examine long-term cellular immune responses to standard and fractional doses of BNT162b2 given as a third dose. A total of 601 participants, primed with ChAdOx1-S, BBIBP-CorV, or Gam-COVID-Vac, were randomized (1:1) to receive 15 μg (fractional dose) or 30 μg (standard dose) of BNT162b2. A subset of participants (N = 256) were enrolled for cell-mediated immunity analysis (n = 101 ChAdOx1-S primed, n = 117 BBIBP-CorV primed, n = 38 Gam-COVID-Vac primed). Antibodies were measured for binding anti-Spike IgG and neutralizing antibodies, and T-cell responses were measured using activation-induced marker (AIM), intracellular cytokine staining (ICS), and IFN-γ against SARS-CoV-2 Spike-specific wild-type and JN.1, and followed-up for 12 months. At 12 months post-vaccination, wild-type and JN.1 IgG levels were sustained and remained approximately 1.7–2.7-fold higher than baseline levels, and neutralizing antibodies were maintained (89% inhibition) for each priming stratum and standard and fractional dose groups. Across all study visits, total AIM CD4mem (expressing CD69+, OX40+, or CD137+) and CD8mem (CD69+CD137+), total ICS CD4mem and CD8mem (IL-2+, TNF-α+, or IFN-γ+), and ELISpot IFN-γ remained similar by study arm and priming strata for wild-type and JN.1 responses. CD4mem AIM responses peaked 6 months post-vaccination; by 12 months, responses to wild-type were maintained, whereas JN.1 responses had declined to day 28 levels or lower. For memory T-cell ICS responses, durability was maintained over 12 months post-vaccination for both wild-type and JN.1 responses. Overall, fractional booster dosing produced comparable and robust long-term humoral and cellular immune responses to a standard dose, including against the JN.1 variant.

## 1 Introduction

COVID-19 booster vaccines play a crucial role in broadening protection against emerging SARS-CoV-2 variants (1). The Omicron subvariant JN.1 has been the predominant variant of interest since 2024, with sub-lineages NB.1.8.1 and XFG currently on the rise globally (2). With each new dominant variant, there is an associated rise in COVID-19 infections, hospitalizations, and deaths, albeit at a reduced rate compared to older variants, particularly in older adults and immunocompromised individuals (3).

Vaccine-induced antibody responses (IgG and neutralizing) are recognized as the main correlates of protection against symptomatic COVID-19 infection. Cell-mediated immunity (CMI) by T cells elicits more stable and durable protection against emerging variants and is thought to protect against severe COVID-19 disease (4, 5). COVID-19 vaccination stimulates a robust SARS-CoV-2 specific CD4 T-cell response, expressing a range of activation (i.e., OX40, CD137 and CD69) and polyfunctional (i.e., IFNγ, TNFα, and IL-2) markers, with CD4 memory cells still detected after 6–10 months (6–11).

mRNA technology in COVID-19 vaccines has the advantage of being modified rapidly in response to emerging variants, with several updated vaccines (e.g., bivalent ancestral + Omicron BA.1, bivalent ancestral + Omicron BA.4/5, and monovalent XBB.1.5) developed in the last 3 years. The new monovalent KP.2/3 vaccine was approved by the U.S. Food and Drug Administration (FDA) in August 2024 (12). While these updated vaccines are available mostly in high-income countries, COVID-19 booster uptake remains low (13, 14). In contrast, many low- and middle-income countries (LMICs) still use ancestral-based vaccines, with only 32.8% of adults living in LMICs having received at least one COVID-19 vaccine dose (13), although these data are relatively limited in LMICs.

Fractional dosing is an effective method to reduce costs and improve vaccine coverage and has been used successfully for other vaccines (15, 16). For COVID-19 vaccines, fractional dosing reduces vaccine reactogenicity but is still able to induce similar antibody levels in the short- to middle-term (1–8 months) (17–19). However, several knowledge gaps remain, including the duration of antibody and cellular immune protection following fractional-dose vaccination and whether boosting with an ancestral COVID-19 vaccine generates protective immune responses to emerging SARS-CoV-2 variants.

We conducted a randomized controlled trial (RCT) in healthy Mongolian adults to examine the reactogenicity and immunogenicity of a standard (30 μg) and fractional (15 μg) dose of BNT162b2 given as a third dose (first booster). The primary outcomes of this study have been published previously, showing that a fractional dose generated non-inferior antibody responses to the standard dose 28 days post-booster and had lower reactogenicity (20). Furthermore, we recently reported longitudinal humoral immunity and safety outcomes, finding that a fractional dose booster was non-inferior to a standard dose up to 12 months post-booster (21).

In this study, we extend these findings to comprehensively examine the cellular immune responses to the ancestral strain and the JN.1 variant over the 12-month follow-up period following fractional or standard BNT162b2 booster vaccination.

## 2 Methods

### 2.1 Study design

This was a double-blind RCT to determine the immunogenicity of fractional and standard booster (third) doses of the BNT162b2 COVID-19 vaccine in Mongolian adults, as described previously (20). Briefly, participants aged ≥18 years who had received two doses of ChAdOx1-S (ChAd, AstraZeneca), BBIBP-CorV (BBIBP, Sinopharm), or Gam-COVID-Vac (Gam, Sputnik) as their primary series were randomly assigned (1:1) to receive a 15 µg (fractional) or 30 µg (standard) dose of BNT162b2 (Pfizer–BioNTech) as a third (booster) dose, stratified by age group (18–49 years vs ≥50 years) and primary vaccine type. Study staff involved in administering the vaccine were not blinded. Participants were blinded to the allocation until the Day-28 visit, while laboratory staff were blinded during the analysis of specimens. Participants were followed for 12 months post-booster. Participants were asked to test for SARS-CoV-2 if they had respiratory symptoms by PCR or rapid antigen test (RAT) throughout the duration of the trial.

A subset (aimed for approximately 40%) of participants from each group with age-matched strata (<50 and ≥50 years) were included in the cell-mediated immunity (CMI) sub-analysis. Only 10–20 CMI samples were processed per day; therefore, the first 10–20 participants recruited each day were included in the CMI subset. Participants in the CMI subset are the focus of this study.

This study was approved by the Royal Children’s Hospital Human Research Committee (HREC 81800/RCHM-2021) and the Mongolian Ethics Committee of the Ministry of Health (Decision #273, 5 April 2022) (ClinicalTrials.gov, Identifier: NCT05265065; https://clinicaltrials.gov/study/NCT05265065). Written informed consent was obtained from all participants prior to enrolment. The study protocol and eligibility criteria have been previously published (20).

### 2.2 Procedures

#### 2.2.1 Sample processing

Blood samples (30 mL) were taken at baseline (day 0), day 28, 6-, and 12-months post-vaccination and 5 mL of blood was processed for serum and stored at −80 °C. An aliquot of blood (5 mL) was used for the QuantiFERON Human Interferon gamma (IFN-γ) SARS-CoV-2 assay (Qiagen, Hilden, Germany). The remaining blood (20 mL) was processed for peripheral blood mononuclear cells (PBMCs) and stored in liquid nitrogen. Detailed methods have been reported previously and are included in the **Supplementary Methods** (20).

#### 2.2.2 Spike-specific IgG binding assays

Binding IgG antibodies were measured for ancestral (wild-type) Spike-specific IgG using the commercial Euroimmun S1 IgG ELISA (EUROIMMUN Medizinische Labordiagnostika AG, Lübeck, Germany). Omicron subvariant JN.1 Spike-specific IgG were measured using an in-house ELISA (5). The binding IgG data using the Euroimmun S1 IgG ELISA kits (wild-type) are reported as relative units/mL (RU/mL) and converted to binding antibody units (BAU/mL) per the manufacturer’s instructions (multiplication factor = 3.2). Results >35.2 BAU/mL were considered positive. Results for the JN.1 ELISA were presented as ELISA units/mL (EU/mL), with results ≥1.5 EU/mL considered positive.

#### 2.2.3 Surrogate virus neutralization assays

Functional antibodies were measured using a semi-quantitative surrogate virus neutralization test (sVNT) (GenScript cPass SARS-CoV-2, New Jersey, USA) for wild-type, according to the manufacturer’s instructions, and reported in percent inhibition, as previously described (20). A result ≥30% inhibition was considered positive.

#### 2.2.4 Activation induced marker assay and intracellular cytokine staining assays

PBMCs were thawed in 10 mL of R10 media and centrifuged at 600*g* for 3 min at 4 °C. Supernatants were discarded, and after another wash with R10 media, PBMCs were resuspended to a concentration of 2.5 × 10^6^ cells/mL and seeded into a 96-well plate (2.5 × 10^5^ cells/well). For the activation-induced marker (AIM) assay, PBMCs were stimulated with 1 μg/mL wild-type (PepTivator SARS-CoV-2 Prot_S Com, Miltenyi Biotec, North Rhine-Westphalia, Germany) or 1 μg/mL JN.1 (PepMix™ SARS-CoV-2, JPT Technologies, Berlin, Germany) Spike-specific peptides. For the intracellular cytokine staining (ICS) assay, 2.5 × 10^6^ cells/mL PBMCs were stimulated with 2 μg/mL wild-type or JN.1 peptides and incubated for 2 h at 37 °C. Then, 5 µg/mL Brefeldin A solution (Biolegend, San Diego, California, United States) was added to the cells. Unstimulated controls included peptide buffers: R10 media (wild-type) and DMSO (JN.1). PBMCs were incubated for 24 h at 37 °C with 5% CO_2_. After 24 h, supernatants from the AIM assay were collected for cytokine analysis.

PBMCs were washed in flow buffer (PBS + 2% FBS), and cells were stained with the respective antibody panels (**Supplementary Table 1**). For ICS assays, fixation buffer (BD Biosciences, Franklin Lakes, New Jersey, United States) was added after ICS Staining Panel 1 and incubated at 4 °C for 20 min, followed by washing in permeabilization buffer (BD) subsequent staining with ICS Staining Panel 2 (**Supplementary Table 1**). After antibody staining, PBMCs were resuspended in 120 μL of flow buffer and a minimum of 2 × 10^5^ cells were captured on the Cytek Aurora spectral flow cytometer (Cytek, Fremont, California, United States) and analyzed using FlowJo Software v10 (Ashland, Oregon, United States). After background correction (stimulation minus unstimulated), activation marker and cytokine expression were low; therefore, an OR Boolean gating strategy was utilized to present results as the frequency of total AIM (the sum of each marker expression for CD69^+^, OX40^+^, OR CD137^+^), and total ICS (cytokine-expressing cells, the sum of each marker expression for IL-2, TNF-α OR IFN-γ) for memory CD4 and memory CD8 T cell populations. The gating strategy for the AIM and ICS assays is shown in **Supplementary Figure 1**.

#### 2.2.5 Measurement of interferon-gamma producing cells

A SARS-CoV-2 IFN-γ ELISpot was conducted on a random subset of 114/256 (44.5%) participants to measure the number of IFN-γ producing cells (22). Briefly, 96-well Multiscreen^®^ filter plates (Merck Millipore) were coated with anti-human IFN-γ capture antibody (5 μg/ml; BD Bioscience; San Diego, CA) and stored overnight at 4 °C. The plate was then washed three times with PBS and blocked with R10 media for 2 h at room temperature. PBMCs (2 × 10^5^ cells/well) were stimulated with wild-type and JN.1 Spike-specific peptides (1 μg/mL), positive control (phytohemagglutinin; PHA, 2 μg/mL; ThermoFisher Scientific), or left unstimulated (R10 media and DMSO), for 16 h–18 h at 37 °C with 5% CO_2_. ELISpot plates were developed using biotinylated anti-human IFN-γ detection antibody, streptavidin–horseradish peroxidase (1:100), and substrate solution (3-Amino-9-ethylcarbazole (AEC)), according to the manufacturer’s instructions (BD Bioscience; San Diego, CA). Plates were read and analyzed using an automated ELISpot reader and software version 6.0 (AID GmbH, Strassberg, Germany). Results are displayed as IFN-γ spot-forming units (SFU) per 10^6^ cells.

Interferon gamma (IFN-γ) concentration was also measured on the full CMI subset (n = 256) using the QuantiFERON Human Interferon gamma SARS-CoV-2 kit which have been reported previously (23). Detailed methods are provided in the **Supplementary Methods**.

#### 2.2.6 Multiplex cytokine assay

Supernatants collected from the AIM assay were used to measure IL-2, IL-4, IL-6, IFN-γ, and TNF-α in a multiplex bead-based assay (Bio-Rad Laboratories, Hercules, California, United States), following the manufacturer’s instructions. Results are presented in pg/mL after subtraction of values from unstimulated control conditions.

#### 2.2.7 Documented and undocumented SARS-CoV-2 infections

Documented SARS-CoV-2 infections were defined as participant-reported positive PCR or rapid antigen tests, while undocumented infections were defined as a ≥1.2-fold rise in wild-type Spike-specific IgG levels between visits (day 28–6 months and 6–12 months). This classification to detect undocumented BTI (≥1.2 fold rise), was based on a conservative threshold derived from unpublished data obtained from our Melbourne study (24), and similar studies (21, 25, 26).

The co-primary outcomes of this RCT have been published (20) and included reporting on binding antibodies measured at 28 days post-booster, reactogenicity within one week post-booster, and any adverse or serious adverse events. The secondary outcomes presented here report on the CMI subset only and include binding IgG antibodies to wild-type and JN.1, neutralizing antibodies (sVNT) against wild-type, and CMI responses measured using an IFN-γ-release assay (ELISpot) and high-dimensional flow cytometry to assess the frequency of total AIM (CD69+, OX40+, and CD137+) and total ICS (IL-2, TNF-α and IFN-γ) in memory CD4 and memory CD8 T-cell populations. These immune outcomes were measured at baseline, day 28, and 6- and 12-month post-booster timepoints.

### 2.3 Statistical analysis

The statistical plan and methods used for this study have been previously detailed (20). Analyses included all randomized participants as per randomization with complete outcome and covariate data, irrespective of post-randomization SARS-CoV-2 infection. Binding antibody data are presented as geometric mean concentrations (GMC) and 95% confidence intervals (CI). A geometric mean ratio (GMR) was used for comparison between fractional and standard doses (fractional/standard) at each time point within each priming stratum for all antibody data. Linear regression was used to estimate the differences in log antibody concentrations, adjusted for age group, baseline levels, priming strata, timing of blood draw, and dosing intervals. The GMR was calculated as the antilogarithm of the mean difference between the vaccine dose groups with a 95% CI. sVNT and IFN-γ data from the QuantiFERON and ELISpot assays are presented as a median and interquartile range (IQR), with comparisons between standard and fractional dose groups at each timepoint were made using a Mann–Whitney U-test. All flow cytometric and cytokine data were presented as a GMC with 95% CI, and comparisons between standard and fractional dose groups at each time point were made using a parametric t -test on log-transformed data. Comparison across time points were made using a paired Wilcoxon signed-rank test. Comparisons of undocumented suspected SARS-CoV-2 infections were measured by study arms and priming strata using a chi-squared test. Although the primary endpoint of this trial was analyzed under a non-inferiority framework (20), analyses in this study are based on a superiority framework, as specified in the statistical analysis plan. Analyses were performed using Stata software version 19 (College Station, TX: StataCorp LLC) and GraphPad Prism v10 (Boston, Massachusetts, United States).

## 3 Results

**Figure 1:**
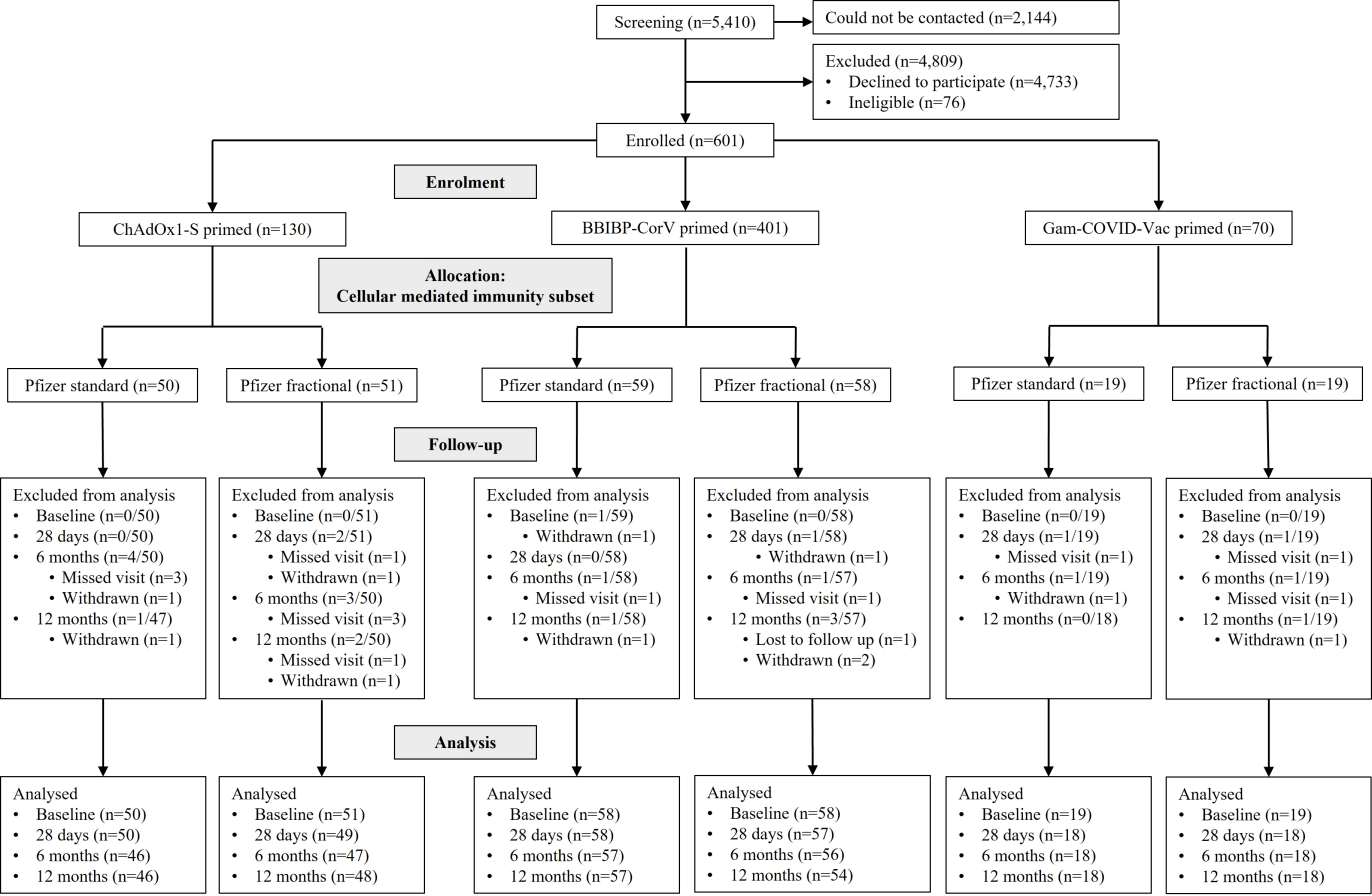
Trial profile. A total of 5,410 potential participants were screened, with a total of 601 participants that met eligibility criteria and were enrolled into the study, for randomization/allocation. A subset of 256 participants were allocated to the cellular mediated immunity (CMI) subset.

Between 27 May and 30 September 2022, 5,410 individuals were screened and 601 enrolled. Of these, 256 (42%) were enrolled into the CMI subset used for this analysis (**Figure 1**). CMI subset participants were distributed across priming groups as follows: ChAdOx-1 (standard dose n = 50; fractional dose n = 51), BBIBP-CorV (standard n = 59; fractional n = 58), and Gam-COVID-Vac (standard n = 19; fractional n = 19). Study retention at 12 months was 94.1% (241 of 256).

### 3.1 Baseline characteristics

The baseline characteristics of the study participants enrolled in the CMI subset (**Table 1**) were similar to the overall cohort reported previously (**Supplementary Table 2**) (20). The median age was similar by study arm (standard, 36 years (IQR 31–51); fractional, 41 years (IQR 33–51)), which was slightly younger than that of the whole study cohort (standard, 44 years (IQR 32–55); fractional, 44 years (IQR 33–55)). There was a total of 180 (70.3%) participants aged ≥18 to 49 years and 76 (29.7%) participants aged ≥50 years, with similar distribution within each COVID-19 vaccine priming stratum, which was more skewed toward the younger age group than the whole study cohort of 60.2% and 39.8%, respectively. Baseline characteristics were balanced by design (age group and priming strata). Small chance imbalances were observed in sex and smoking within the Gam-COVID-Vac stratum; models adjusted for dosing intervals and age, which were very similar to the overall study cohort. Additional adjustment for sex and smoking was considered, but given the small cell counts within this stratum and the absence of other material imbalances, further adjustment was not undertaken.

**Table 1:** Baseline characteristics by study group allocation.

Baseline characteristics	All priming strata			ChAd-primed		BBIBP-primed		Gam-primed	
	Total	Standard	Fractional	Standard	Fractional	Standard	Fractional	Standard	Fractional
	N = 256	N = 128	N = 128	N = 50	N = 51	N = 59	N = 58	N = 19	N = 19
Participant age at enrolment	40 (32–51)	36 (31–51)	41 (33–51)	34 (32–41)	40 (34–48)	43 (26–55)	42 (31–54)	42 (28–53)	41 (32–52)
Age group (%)									
<50 years	180 (70·3%)	91 (71·1%)	89 (69·5%)	43 (86·0%)	41 (80·4%)	35 (59·3%)	35 (60·3%)	13 (68%)	13 (68%)
≥50 years	76 (29·7%)	37 (28·9%)	39 (30·5%)	7 (14·0%)	10 (19·6%)	24 (40·7%)	23 (39·7%)	6 (32%)	6 (32%)
Participant sex									
Male	131 (51·2%)	64 (50·0%)	67 (52·3%)	28 (56·0%)	26 (51·0%)	26 (44·1%)	27 (46·6%)	10 (53%)	14 (74%)
Female	125 (48·8%)	64 (50·0%)	61 (47·7%)	22 (44·0%)	25 (49·0%)	33 (55·9%)	31 (53·4%)	9 (47%)	5 (26%)
BMI, Kg/m^2^	25·5 (23·0–29·4)	25·5 (23·0–29·3)	25·5 (23·0–29·4)	27·2 (23·7–29·7)	26·2 (23·2–29·4)	24·7 (21·2–28·7)	24·8 (22·7–28·7)	25·2 (23·0–29·4)	25·8 (24·1–29·4)
Self-reported SARS-CoV-2 infection before study commencement	147 (57·4%)	73 (57·0%)	74 (57·8%)	32 (64·0%)	35 (68·6%)	29 (49·2%)	25 (43·1%)	12 (63%)	14 (74%)
Days between 1st and 2nd doses	40 (28–46)	40 (28–45)	39 (28–48)	42 (41–47)	43 (40–48)	28 (25–33)	28 (24–31)	52 (41–64)	61 (55–72)
Days between 2nd dose and study (3rd) dose	430 (398–506)	437 (388–508)	427 (401–506)	508 (476–524)	510 (471–522)	400 (371–446)	405 (391–430)	424 (368–444)	418 (389–431)
Comorbidities									
Diabetes mellitus	10 (3·9%)	8 (6·2%)	2 (1·6%)	2 (4·0%)	1 (2·0%)	4 (6·8%)	0 (0·0%)	2 (11%)	1 (5%)
Cardiovascular disease	20 (7·8%)	9 (7·0%)	11 (8·6%)	1 (2·0%)	5 (9·8%)	6 (10·2%)	5 (8·6%)	2 (11%)	1 (5%)
Hypertension	60 (23·4%)	27 (21·1%)	33 (25·8%)	10 (20·0%)	10 (19·6%)	14 (23·7%)	16 (27·6%)	3 (16%)	7 (37%)
Chronic kidney disease	16 (6·2%)	7 (5·5%)	9 (7·0%)	0 (0·0%)	2 (3·9%)	4 (6·8%)	6 (10·3%)	3 (16%)	1 (5%)
Cigarette user	69 (27·0%)	34 (26·6%)	35 (27·3%)	15 (30·0%)	13 (25·5%)	10 (16·9%)	16 (27·6%)	9 (47%)	6 (32%)
Currently pregnant	0 (0·0%)	0 (0·0%)	0 (0·0%)	0 (0·0%)	0 (0·0%)	0 (0·0%)	0 (0·0%)	0 (0·0%)	0 (0·0%)

Data are median (IQR) or n (%). No data were missing for the variables reported.

### 3.2 Spike-specific IgG binding response

**Figure 2:**
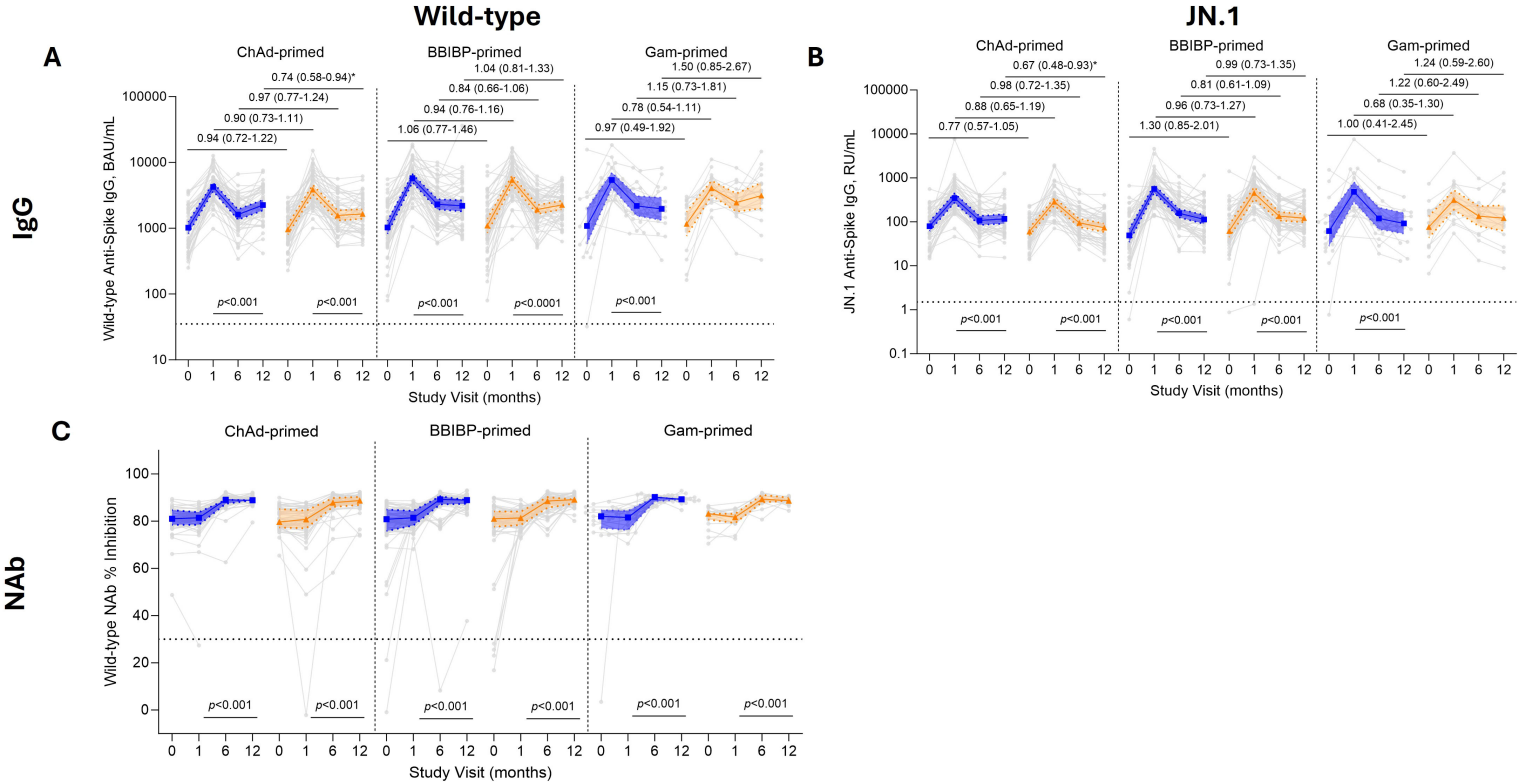
Binding and sVNT responses up to 12 months following fractional (orange) or standard (blue) dose booster vaccination. The top panel displays binding IgG data for wild-type **(A)** and JN.1 **(B)**. The bottom panel displays functional antibody as measured by a surrogate virus neutralizing test (sVNT) for wild-type **(C)**. IgG results are displayed as GMC ± 95% CI, with fractional dose/standard dose comparison results are displayed with the geometric mean ratio (GMR) and 95% CI at all time points. sVNT data is presented as the percent inhibition with median ± IQR, with a Mann–Whitney U test was used to compare between standard and fractional dose groups. Both IgG and sVNT data are presented with a *p*-value for paired Wilcoxon-signed-rank test between day 28 and 12 months post-vaccination. The GMR was adjusted for age group, baseline levels, priming strata, timing of blood draw, and dosing intervals. Horizontal lines indicate seropositivity cut-off for each assay platform and variant. BAU, binding antibody units; RU, relative units.

At baseline, wild-type SARS-CoV-2 Spike IgG levels were similar by study arm and priming strata (**Figure 2A**; **Supplementary Table 3**). At day 28 post-vaccination, the wild-type Spike-specific IgG increased across priming strata by 4.2–5.6-fold in the standard dose groups, and by 3.5–4.9-fold in the fractional dose groups. Similar GMRs were found between standard and fractional dose groups in the ChAd- (0.90 [95% CI 0.73–1.11]) and BBIBP-primed arms (0.94 [95% CI 0.76–1.16]), whereas a lower GMR was observed for the Gam-primed arm (0.78 [95% CI 0.54–1.11]) that still crossed the null value. At 6 months post-vaccination, IgG concentrations waned across all priming strata, yet the GMR remained similar by study arm (ChAd-primed: GMR of 0.97 [95% CI 0.77–1.24], BBIBP-primed: GMR of 0.84 [95% CI 0.66–1.06], and Gam-primed: GMR of 1.15 [95% CI 0.73–1.81]). At 12 months post-vaccination the IgG levels were maintained, with a similar GMR among the different priming strata, except among ChAdOx-1-primed participants (GMR 0.74 [95% CI 0.58–0.94]).

For the JN.1 variant, baseline Spike-specific IgG was similar between standard and fractional dose groups and across all priming strata (**Figure 2B**, **Supplementary Table 3**). At 28 days post-vaccination, Spike-specific IgG increased 4.5–12.7-fold in the standard dose group, and 4.5–9.0-fold in the fractional dose groups, with the greatest increase observed in BBIBP-primed participants. As with Spike-specific IgG for wild-type, a similar pattern was observed for JN.1 by priming strata; GMRs of 0.88 (95% CI 0.65–1.19) for ChAd- and 0.96 (95% CI 0.73–1.27) for BBIBP-primed participants, whereas Gam-primed participants who received a fractional dose had lower antibody levels than the standard dose group (GMR of 0.68 [95% CI 0.35–1.30]). At 6 months post-vaccination, JN.1 IgG concentrations waned across all priming strata, and the GMR remained similar by study arm and priming strata (ChAd-primed: GMR of 0.98 [95% CI 0.72–1.35], BBIBP-primed: GMR of 0.81 [95% CI 0.61–1.09], Gam-primed: GMR of 1.22 [95% CI 0.60–2.49]). At 12 months post-vaccination, IgG levels were maintained with a similar GMR among the different priming strata, except among ChAd-primed participants (GMR of 0.67 (95% CI 0.48–0.93]). Overall, the wild-type and JN.1 IgG response at 12 months post-vaccination was approximately 1.7–2.7-fold higher than baseline for wild-type IgG values, and approximately 1.2–2.6-fold higher for JN.1-specific IgG.

### 3.3 Surrogate virus neutralization response

At baseline, wild-type sVNT responses were similar between standard and fractional dose groups across all priming strata, with a median percent inhibition range of 80%–83% (**Figure 2C**; **Supplementary Table 4**). At 28 days post-booster, sVNT responses remained stable among all standard and fractional dose groups and priming strata, with a median percent inhibition of 81%–82% inhibition. At 6 months post-vaccination, median percent inhibition increased to 88%–90%, which was maintained until 12 months post-vaccination between standard and fractional groups and priming strata. We were unable to measure JN.1 sVNT responses as the reagents were not available at the time of analysis.

### 3.4 T cell memory response by activation-induced marker assay

**Figure 3:**
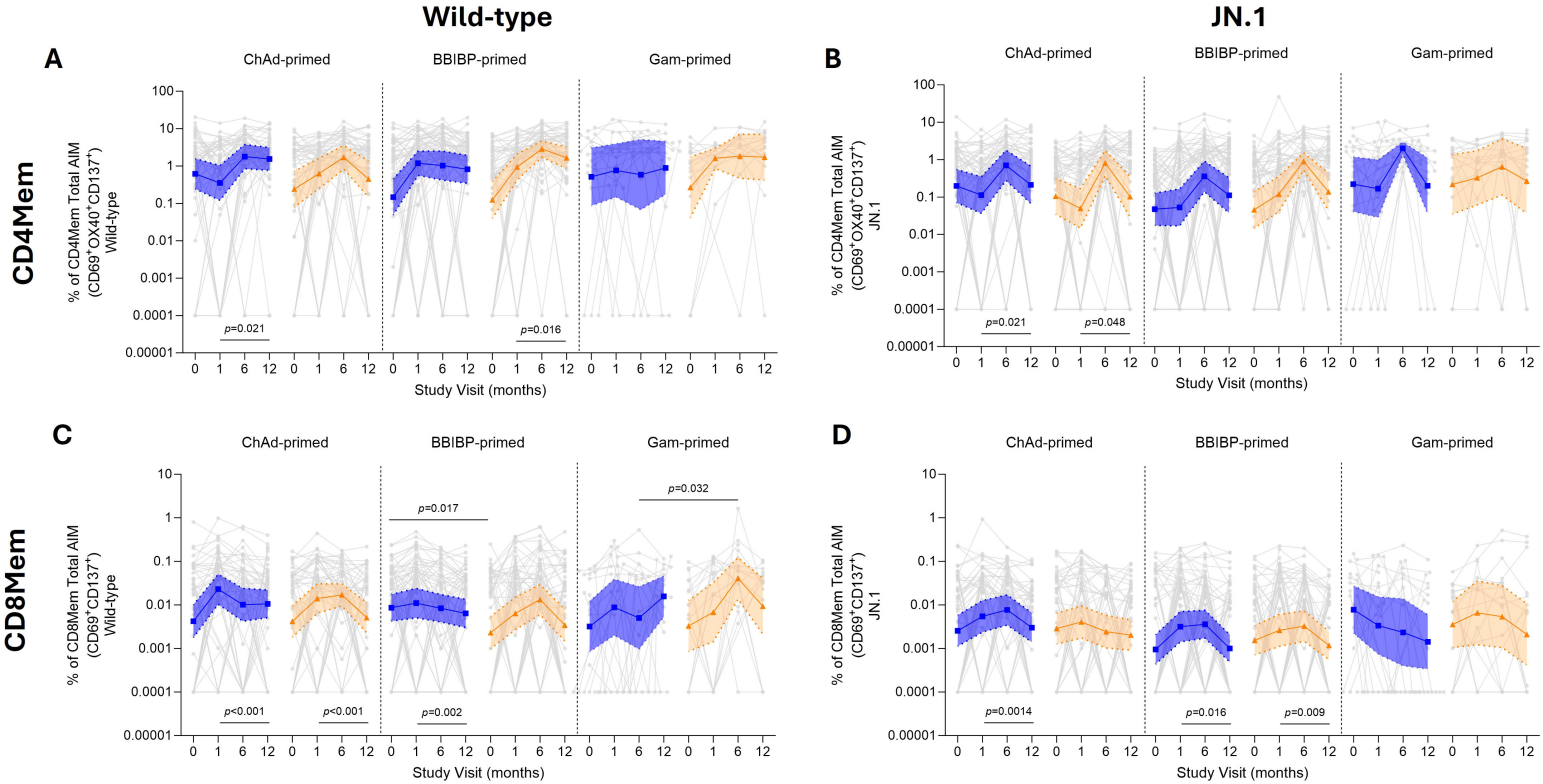
T cell memory responses by AIM assay up to 12 months following fractional (orange) or standard (blue) dose booster vaccination. Total CD4mem AIM (CD69^+^OX40^+^CD137^+^) for wild-type **(A)** and JN.1 **(B)**. Total CD8mem AIM (CD69^+^CD137^+^) for wild-type **(C)** and JN.1 **(D)**. Results displayed were background subtracted using the respective control groups for wild-type (media) and JN.1 (DMSO), with results shown as GMC ± 95% CI. Data was log transformed, and a parametric t-test was used to compare between standard and fractional dose groups. A paired Wilcoxon signed-rank test was done comparing day 28 and 12 months post-vaccination.

For activation of memory T cells using the AIM assay with both wild-type and JN.1, there was a strong stimulatory response compared with their respective unstimulated controls (media or DMSO controls, respectively) (**Supplementary Figure 2**). AIM responses did not differ between standard and fractional dose groups and priming strata at all time points, for wild-type and JN.1, for CD4mem (expressing CD69^+^, OX40^+^ or CD137^+^) and CD8mem (CD69^+^CD137^+^) total AIM responses (**Figures 3A, B**; **Supplementary Table 5**).

For CD4mem, AIM responses peaked at 6 months post-vaccination for wild-type, with ~7-fold higher levels from baseline for all groups, except for the BBIBP-primed fractional dose participants, where a 23-fold increase from baseline was observed. However, the BBIBP-primed and the Gam-primed participants who received the standard booster dose peaked at 28 days post-booster (BBIBP-primed: 8-fold rise, Gam-primed: 1.5-fold rise) (**Supplementary Table 5**). A similar pattern was observed for JN.1, with the peak AIM response at 6 months post-vaccination across all groups with a ~2.9–9-fold rise from baseline levels (**Figure 3B**), except for the BBIBP-primed participants following a fractional dose (~20-fold increase). At 12 months, AIM responses had stabilized for wild-type (except for ChAd-primed participants in the fractional dose group, whose levels had waned), whereas for JN.1, CD4Mem AIM responses had waned to levels comparable to day 28.

CD8mem AIM responses were similar between standard and fractional doses at each time point for both wild-type and JN.1 (**Figures 3C, D**; **Supplementary Table 5**). At day 28 post-booster, CD8mem AIM responses for wild-type increased ~1.2–5.8-fold from baseline. Similarly, for JN.1, at day 28, all groups had GMC increases of 1.3–3-fold from baseline, except for Gam-primed participants following a standard dose (2.6-fold decrease). However, peak wild-type AIM CD8mem responses occurred at 6 months for BBIBP-primed participants (6.5-fold rise from baseline) and Gam-primed participants (13.3-fold rise from baseline) following a fractional dose, while Gam-primed participants following a standard dose peaked at 12 months (5.3-fold rise from baseline). At 12 months post-vaccination, wild-type and JN.1 CD8mem AIM responses waned to baseline for most groups.

### 3.5 T cell memory response by intracellular cytokine staining

**Figure 4:**
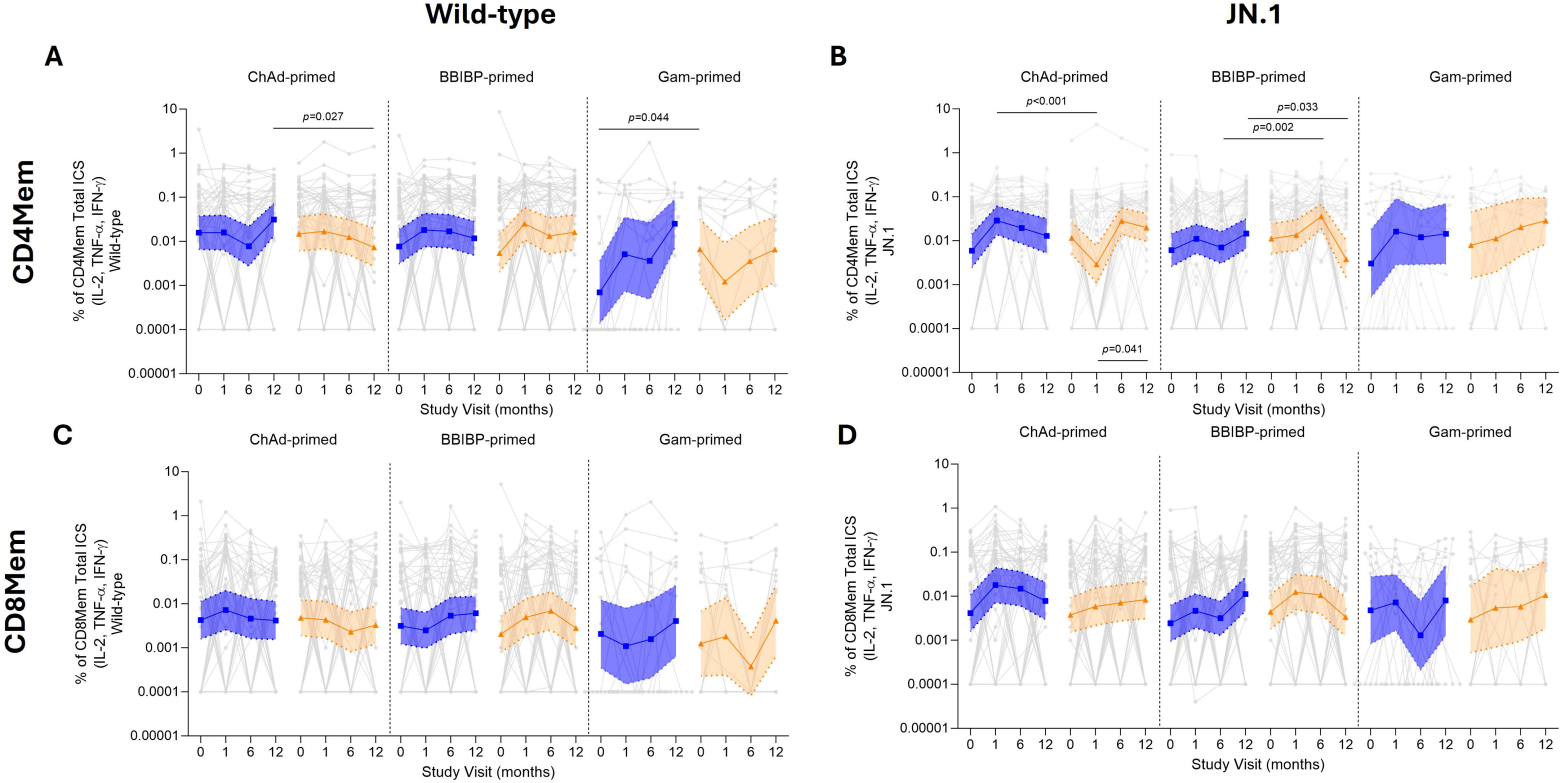
T cell memory responses by ICS assay up to 12 months following fractional (orange) or standard (blue) dose booster vaccination. Total CD4mem ICS (IL-2^+^TNF-α^+^IFN-γ^+^) for wild-type **(A)** and JN.1 **(B)**. Total CD8mem ICS (IL-2^+^TNF-α^+^IFN-γ^+^) for wild-type **(C)** and JN.1 **(D)**. Results displayed were background subtracted using the respective control groups for wild-type (media) and JN.1 (DMSO), with results shown as GMC ± 95% CI. Data was log transformed, and a parametric t-test was used to compare between standard and fractional dose groups, with a *p* value shown if significant. A paired Wilcoxon signed-rank test was done comparing day 28 and 12 months post-vaccination.

ICS responses were similar between standard and fractional dose groups and priming strata for memory T cell ICS (IL-2^+^, TNF-α^+^, or IFN-γ^+^) responses to wild-type and JN.1 stimulation (**Figure 4**; **Supplementary Table 6**). Overall, both wild-type and JN.1 had a strong stimulatory response relative to their unstimulated responses (media and DMSO, respectively) (**Supplementary Figure 3**).

For wild-type, total CD4mem ICS (**Figures 4A, B**) were similar between standard and fractional dose groups, apart from ChAd-primed at 12 months. Similarly, for JN.1, there were no differences between standard and fractional dose groups for most priming strata across all time points, except for the ChAd-primed group at day 28, and the BBIBP-primed group at 6-months. However, for CD4mem, ICS responses against wild-type were stable over time for all groups, except Gam-primed participants following a standard dose, who displayed a 25-fold-rise from baseline at 12 months post vaccination. For JN.1, CD4mem ICS responses increased across all groups at 28 days post-booster by 1.2–5.3-fold, apart from ChAd-primed participants, whose responses waned by 4-fold.

For CD8mem ICS responses (**Figures 4C, D**), no differences were observed between standard and fractional dose groups over the 12-month period post-booster for both wild-type and JN.1, except for JN.1 in the BBIBP-primed group at day 28 and 6 months. Across all groups, CD8mem ICS responses remained stable over 12 months post-vaccination for both wild-type and JN.1. An exception to this was Gam-primed participants at 6 months; responses waned by 1.75-fold and 4-fold for wild-type in the fractional and standard dose groups, respectively.

### 3.6 ELISpot IFN-γ response

**Figure 5:**
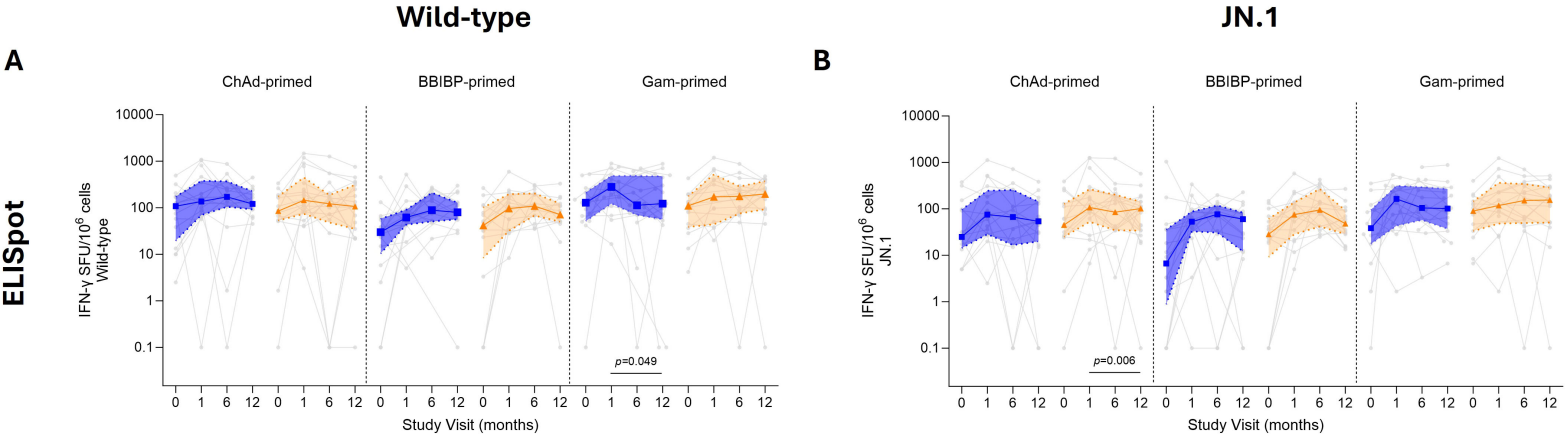
IFN-γ producing cell responses by ELISpot up to 12 months following fractional (orange) or standard (blue) dose booster vaccination. IFN-γ spot-forming units (SFU) per 10^6^ PBMCs for wild-type **(A)** and JN.1 **(B)**. Results are shown as median ± IQR. A Mann–Whitney U test was used to compare between standard and fractional dose groups, with a *p* value shown if significant. A paired Wilcoxon signed-rank test was done comparing day 28 and 12 months post-vaccination.

IFN-γ producing T cells measured by ELISpot were similar between standard and fractional doses for wild-type and JN.1 across all time points (**Figures 5A, B**; **Supplementary Table 7**). For both wild-type and JN.1, the number of IFN-γ producing T cells steadily increased over 12 months post-booster vaccination in all groups. At 12 months, wild-type IFN-γ T cell responses in the ChAd-primed participants increased from baseline by 1.1–1.3-fold, BBIBP-primed participants increased by 1.7–2.7-fold, except for Gam-primed participants, whose responses waned by 1.8-fold following the fractional dose with no change observed following the standard dose. At 12 months for JN.1, IFN-γ T cell responses in the ChAd-primed participants increased by ~2.2-fold from baseline, which was similar to Gam-primed participants with a 1.7–2.9-fold rise, while BBIBP-primed participants had a higher fold-rise of ~8.6 from baseline.

For QFN Ag1 (CD4-specific T cell responses) and Ag2 (CD4- and CD8-specific T cell responses), wild-type-specific IFN-γ release at baseline was similar between standard and fractional dose groups, as reported previously (23) (**Supplementary Figure 4**).

### 3.7 Multiplex cytokine response

**Figure 6:**
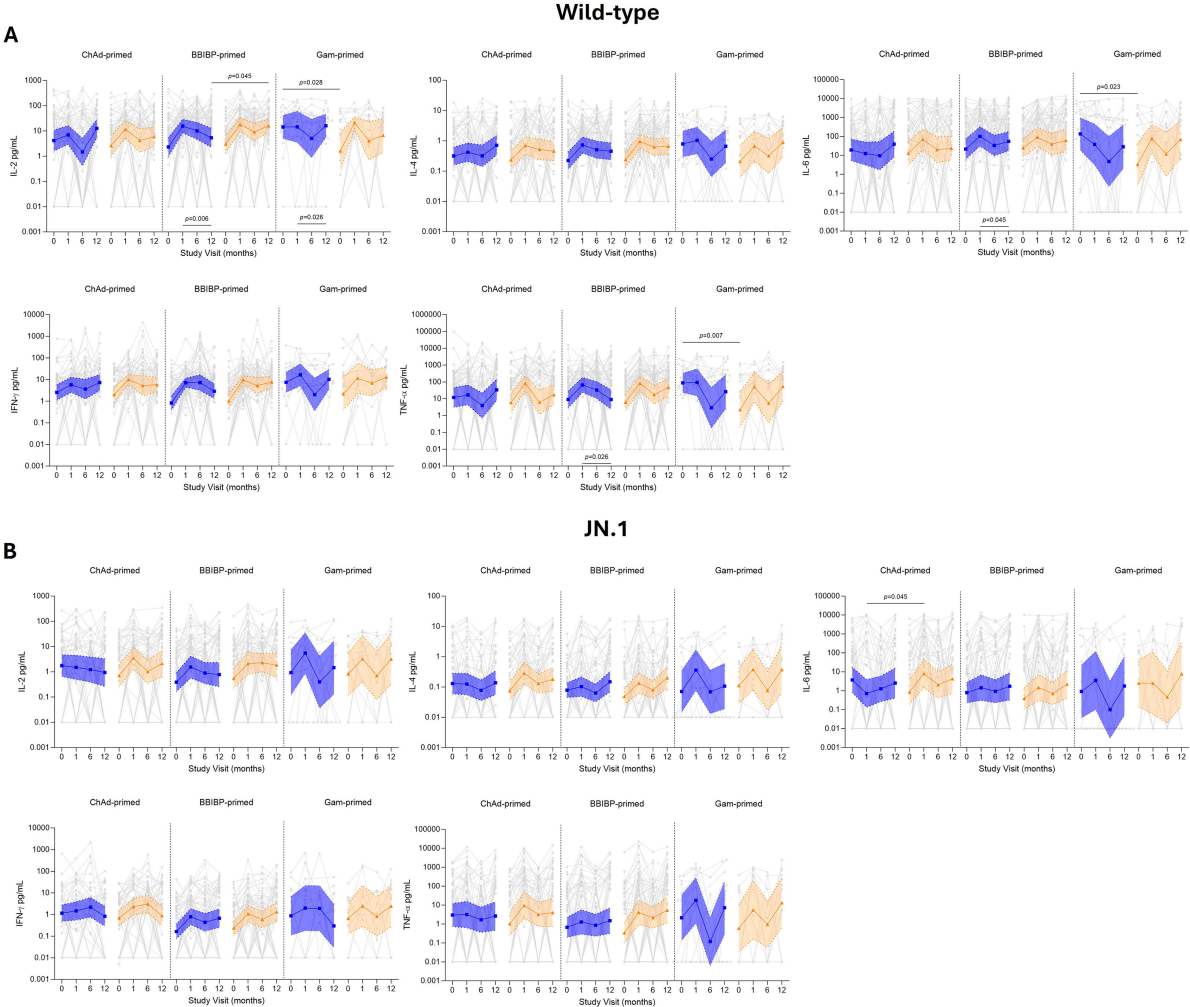
Multiplex cytokine analysis up to 12 months following fractional (orange) or standard (blue) dose booster vaccination. Supernatants collected from the AIM assay were used to measure IL-2, IL-4, IL-6, IFN-γ, and TNF-α secretion for wild-type **(A)** and JN.1 **(B)**. Results are presented in pg/mL, with the unstimulated conditions subtracted from the stimulated conditions. Results are shown as GMC ± 95% CI. Data was log transformed, and a parametric t-test was used to compare between standard and fractional dose groups, with a *p* value shown if significant. A paired Wilcoxon signed-rank test was done comparing day 28 and 12 months post-vaccination.

We assessed the cytokine profile following fractional or standard dose vaccination against wild-type and JN.1 (**Figure 6**; **Supplementary Table 8**). No differences were observed between standard and fractional dose groups or between priming strata for all cytokines examined for wild-type and JN.1, except for IL-6 and TNF-α responses to wild-type in the Gam-primed participants at baseline. At day 28, for wild-type responses most groups responded well following the booster dose for both standard and fractional doses (IL-2: 1.7–13.1-fold rise; IL-4: 1.3–3.9-fold rise; IL-6: 3.7–23.1-fold rise, except for ChAd- and Gam-primed standard dose participants which waned by 1.5 and 3.6-fold, respectively; IFN-γ: 2.2–9.3-fold rise; TNF-α: 1.4–22.9-fold rise). Similarly for JN.1 responses at day 28 most groups responded well to the booster dose, albeit a lower magnitude (IL-2: 3.8–5.9-fold rise; IL-4: 1.3–5.1-fold rise; IL-6: 1.8–9.3-fold rise; IFN-γ: 1.3–4.9-fold rise; TNF-α: 1.1–12-fold rise), with the exception of ChAd-primed participants following a standard dose where levels decreased by 1.1-, 1.2-fold, and 5.2-fold for IL-2, IL-4, and IL-6, respectively. Overall, the cytokine levels stabilized or slightly waned over the 12-month period, and levels remained slightly higher than baseline for both wild-type and JN.1.

### 3.8 Documented and undocumented SARS-CoV-2 infections

Throughout the study period, there were 15 reported, documented SARS-CoV-2 infections, which were excluded from the undocumented SARS-CoV-2 analysis (**Table 2**). Overall, most of the undocumented SARS-CoV-2 infections in the standard and fractional dose groups occurred between 6 and 12 months post-vaccination (43.4% and 35.5%, respectively), with more SARS-CoV-2 infections in the ChAd-primed standard-dose group (24/42; 57.1%) than in the fractional dose group (13/44; 29.5%).

**Table 2:** Undocumented SARS-CoV-2 infections assessed by ≥1.2-fold change increase in anti-Spike IgG levels between study time points.

Priming strata	Fold change ≥1.2 in anti-spike IgG levels without documented SARS-CoV-2 infection n/N^b^ (%)		
	Standard dose	Fractional dose	Chi-squared test
28 days–6 months			
All	3/118 (2.5%)	4/119 (3.4%)	*p* = 0.710
ChAdOx1-S	1/46 (2.2%)	0/47 (0.00%)	*p* = 0.310
BBIBP-CorV	1/55 (1.8%)	1/55 (1.8%)	*p >*0.999
Gam-COVID-Vac	1/16 (5.9%)	3/17 (17.6%)	*p* = 0.287
6 months–12 months			
All	49/113 (43.4%)	39/110 (35.5%)	*p* = 0.227
ChAdOx1-S	24/42 (57.1%)	13/44 (29.5%)	*p* = 0.010
BBIBP-CorV	20/54 (37.0%)	17/48 (35.4%)	*p* = 0.865
Gam-COVID-Vac	5/12 (29.4%)	9/18 (50.0%)	*p* = 0.214

**Figure 7:**
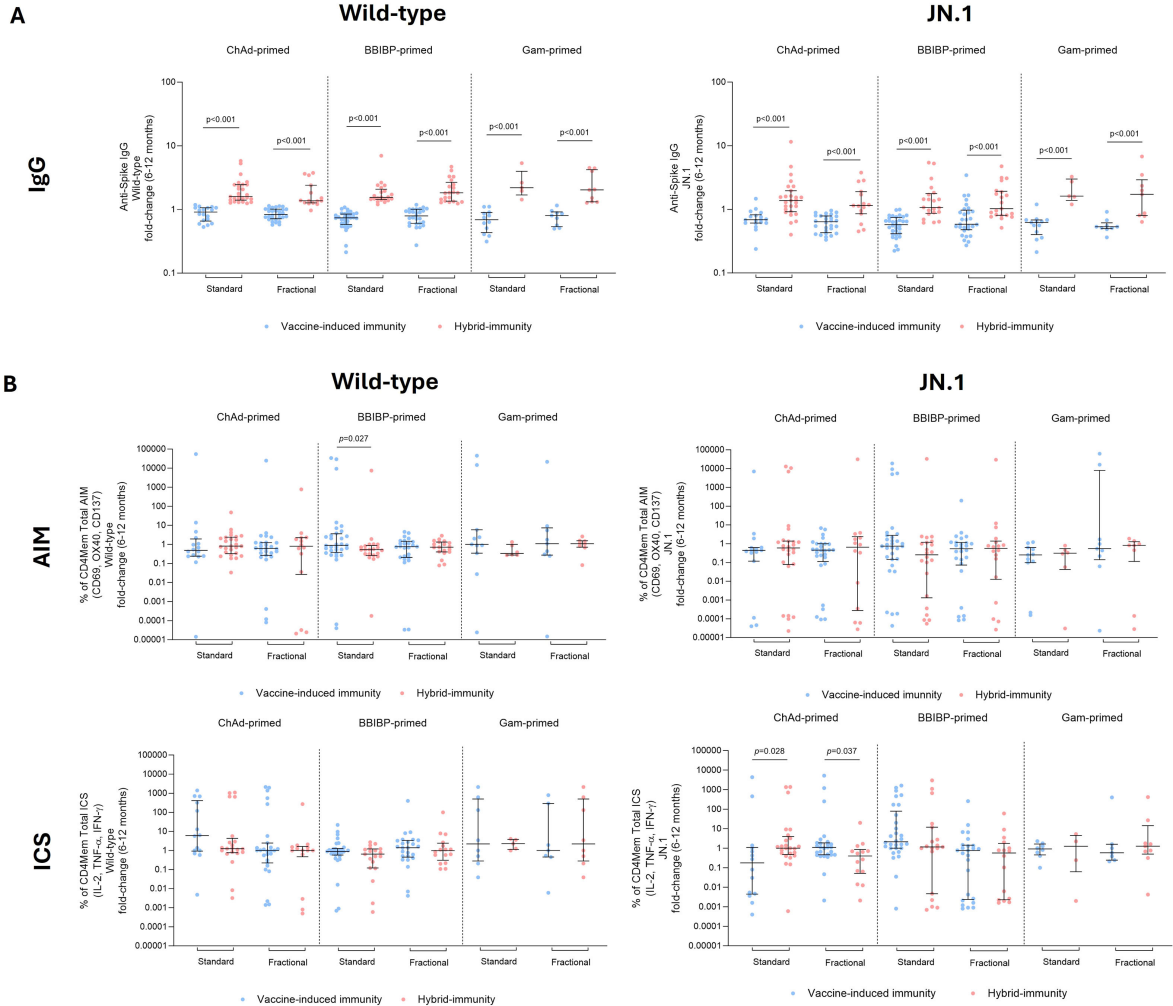
Vaccine-induced immunity (blue) and hybrid-immunity (pink) responses following fractional or standard dose booster vaccination. Vaccine-induced immunity and hybrid-immunity incurred between 6 and 12 months post-vaccine was evaluated for **(A)** IgG and **(B)** CD4Mem AIM (CD60+, OX40+, CD137+) and ICS (IL-2+, TNF-α+, IFN-γ+) responses against wild-type and JN.1. Results are presented as a fold-change (12 months/6 months) post-vaccination as median ± IQR. A Mann–Whitney U test was used to compare between vaccine-induced and hybrid-immunity groups.

Next, we assessed the impact of these documented and undocumented SARS-CoV-2 infections that occurred between 6 and 12 months post-vaccination (**Figure 7**; **Supplementary Figures 5**, **6**). To evaluate vaccine-induced immunity and hybrid immunity between 6 and 12 months post-vaccination, we removed any documented and undocumented infections (suspected BTI, ≥1.2-fold change) in the first 6 months of the study (only seven infections during this period). Here, we found an increase in IgG fold-change between 6 and 12 months post-vaccination between the vaccine-induced immunity and hybrid immunity groups for all priming strata and vaccine arms, against wild-type and JN.1. However, these observations were not seen for CD4mem AIM and ICS T-cell responses, except for ICS responses to the JN.1 variant in the ChAd-primed group. The vaccine-induced and hybrid immunity profiles for IgG and CD4mem AIM T cell responses over the 12-month period are shown in **Supplementary Figures 5**, **6**.

## 4 Discussion

As SARS-CoV-2 variants continue to emerge and escape pre-existing immunity, booster vaccination is recommended to broaden protection (27). However, COVID-19 booster vaccination uptake remains low in many parts of the world (13, 14). Fractional dosing has been proposed as a solution to improve vaccine coverage by reducing vaccine costs and reactogenicity. A key knowledge gap relates to long-term protective immunity following fractional COVID-19 booster vaccination. Our Mongolian RCT is one of the largest studies to date on fractional COVID-19 vaccination, with a 12-month follow-up. In this randomized subset of participants, we report a comprehensive analysis of longitudinal antibody and cellular immunity following a fractional or standard BNT162b2 booster dose, including responses to the JN.1 variant. Overall, we found no differences in serological or cellular immunity between fractional and standard dose groups up to 12 months post-vaccination, with robust and durable responses to JN.1.

Consistent with our previous data, a fractional dose of BNT162b2 produced similar antibody responses over the 12-month follow-up period in this subset of participants (21). Of note, the wild-type binding antibody showed a notable increase in both standard and fractional dose groups at day 28 post-booster dose, while the functional antibody response remained unchanged. This result is not surprising given that the sVNT assay is semi-quantitative and is not able to discriminate responses at high antibody titers. However, we did observe an increase in the Omicron BA.1-specific neutralizing antibody response after vaccination (20). Humoral antibody responses following COVID-19 fractional doses have also been demonstrated previously. A study in Brazil demonstrated that BNT162b2 given as a fractional dose was non-inferior to standard dose following ChAdOx1-S- or BBIBP-CorV-primed individuals by wild-type SARS-CoV-2 Spike-specific IgG levels out to 60 days post-booster (28). Similar BNT162b2 fractional dose non-inferiority findings were observed in a Thailand study following ChAdOx1-S- or CoronaVac-primed individuals up to 20 weeks post-booster (29). A long-term follow-up study to 24 months post-booster dose in Indonesia also demonstrated a non-inferior effect of ChAdOx1-S and BNT162b2 fractional dosing on binding and neutralizing antibodies in ChAdOx1-S- and CoronaVac-primed individuals (25). These studies support the results from our study; however, limited data remain on the cellular immune responses following fractional COVID-19 booster vaccination.

However, our study is one of the first to demonstrate JN.1 IgG responses following ancestral-based vaccination given as a third dose. Our study found that robust JN.1-specific IgG responses were generated by fractional and standard dose BNT162b2, peaking at 28 days post-vaccination and remaining higher than baseline levels at 12 months post-vaccination. Importantly, the majority of the 12-month sera were collected just prior to global JN.1 circulation globally (25 August 2023; only two participants’ 12-month visits were collected after 25 August 2023) (30). Most study participants (57%) self-reported a SARS-CoV-2 infection prior to being recruited into the study (Omicron wave), and this may explain some of the cross-protection found against the JN.1 variant. Other studies have examined JN.1 specific antibody responses following use of updated vaccines, such as mRNA XBB.1, BA.1 or BA.4/5, with outcomes only to one (31–33) or six months post-vaccination (34). Our results are particularly important for many LMICs, which still only have access to the ancestral COVID-19 vaccine.

Our comprehensive analysis of cellular immune responses demonstrated similar responses between the fractional and standard dose groups, regardless of priming vaccine, to both wild-type and JN.1 variants, which were maintained up to 12 months post-vaccination, irrespective of breakthrough infections. Interestingly, our results identified a consistent post-booster effect (day 28 response) for IFN-γ release for both wild-type (QFN and ELISpot assays) and JN.1 (ELISpot assay) responses. These IFN-γ responses 28 days following a fractional dose are also supported by other studies (35, 36). These post-booster effects were observed in our AIM CD4mem and CD8mem T cell responses specific to wild-type strain; however, this was less pronounced in our ICS data. These discrepancies between the AIM and ICS assays are due to different outcome measurements; that is, measuring the response of T cell activation and cytokine secretion. JN.1-specific T cell responses demonstrated a slightly different trend, whereby we observed a post-booster effect at day 28 for AIM CD8mem and ICS CD4mem T cell responses. T cell epitopes from the wild-type strain are highly conserved among newer variants, which explains our observed post-booster T cell response against JN.1 (7, 37, 38). Therefore, despite boosting with the ancestral-based vaccine, both fractional and standard dose boosting with ancestral BNT162b2 demonstrated durable T cell responses to the JN.1 variant.

One explanation for the sustained cellular immune responses following the booster dose is the presence of pre-existing immunity resulting from previous exposure (vaccination and/or infection) (39, 40). A prospective study of 639 participants in Denmark, with a two-year follow-up since the first COVID-19 dose, reported peak cellular responses after three doses, which waned by three months (40). In addition, a recent U.S. study by da Silva Antunes et al., with a cohort of 78 individuals over a 3-year follow-up period showed that after two COVID-19 vaccine doses, T cell responses rapidly plateaued (41). Furthermore, the additive effects of a third or fourth dose were limited, yet contributed to enhanced and stabilized responses providing protection against symptomatic infection (41). These studies highlight the sustained protective effects against severe disease.

Interestingly, we also observed peak CD4mem T cell activation in the AIM assay at 6 months against both wild-type and JN.1 responses, with a greater effect against JN.1. While our low rate of documented SARS-CoV-2 infections (n = 15) was consistent with the low number of cases reported in Mongolia more broadly over the study period (42, 43), using the seroincidence approach (IgG levels ≥1.2-fold rise between study timepoints) (25, 26), we estimated approximately 40% of participants experienced a SARS-CoV-2 infection between the 6- and 12-month time points. This rise in SARS-CoV-2 infections had a similar distribution between the standard and fractional dose groups. While these results add further support to the similar, durable protective effect of fractional dosing compared with standard dosing, our study was not designed to answer this question. Furthermore, we evaluated the differences in humoral and cellular immunity based on documented and undocumented SARS-CoV-2 infections that occurred during the 6- to 12-month study period. Here, we observed distinct differences in antibody responses between vaccine-induced and hybrid-immunity profiles. This was not observed for T-cell responses.

A limitation of this analysis is that microneutralizing antibody data were not available, particularly for JN.1. This study is ongoing and will be reported separately. While our study was strengthened by having cellular immunity data for JN.1, responses to newer variants such as NB.1.8.1 or XFG would also be of interest. Given that these newer variants are similar to JN.1, our results are still highly relevant. The detection of suspected SARS-CoV-2 infection in our analysis, defined as a fold-rise ≥1.2 of wild-type IgG titer without measurement of Nucleocapsid-specific IgG, is a limitation in our study. This was not undertaken because the BBIBP-CorV-primed group, which were vaccinated with an inactivated vaccine containing the N-protein, would have confounded the analysis. Our study cohort was predominantly younger (median 40 years [IQR 32–51]) because older adults had already received a third booster dose at the time of recruitment, and the Gam-primed group had a smaller sample size. Moreover, our results may not be as generalizable to more recent updated COVID-19 variant booster vaccines. Nonetheless, such comprehensive and long-term T cell response data are limited, and many countries still only have access to ancestral-based COVID-19 vaccines.

Overall, our results showed no humoral or cellular immune differences between fractional and standard BNT162b2 administered as a third dose. This demonstrates that a fractional booster dose yields comparable and sustained long-term immune protection to newer variants such as JN.1, similar to a standard dose. In the context of hybrid immunity, fractional doses should be seriously considered to reduce cost and improve vaccine coverage against SARS-CoV-2 in COVID-19 booster strategies. These findings provide novel evidence on the durability and breadth of cell-mediated immunity induced by fractional mRNA booster doses in a real-world LMIC context. 

## Data Availability

The raw data supporting the conclusions of this article will be made available by the authors, without undue reservation.
