# Supplementary material for: Cellular immune responses 12 months after fractional or standard dose BNT162b2 booster vaccination in Mongolian adults
Source: Front Immunol. 2026 Apr 22;17:1779435. doi: 10.3389/fimmu.2026.1779435 (PMC13144050; doi:10.3389/fimmu.2026.1779435)
Supplement: Supplementary file 1 [file Table1.docx]

Supplementary Material

# Supplementary Methods

## Sample processing

Briefly, whole blood was overlaid onto 15mL of Lymphoprep (STEMCELL Technologies, Vancouver, Canada) and centrifuge at 800g for 20 minutes at room temperature with no breaks, a white buffy was collected and washed with RPMI-1640 (Sigma-Aldrich, St. Louis, Missouri, United States) twice (500g for 10 minutes), stored in 1:1 R10 media (RPMI, 10% FBS, 1% p/s, and 1% Glutamax, Sigma-Aldrich) and cold freeze mix (heat inactivated-FBS containing 15% (v/v) DMSO, Sigma-Aldrich) at 8-10x10^6 cells/mL.

## Whole blood interferon-gamma assays

Interferon gamma (IFN-γ) concentration was measured using the QuantiFERON Human Interferon gamma SARS-CoV-2 kit according to the manufacturer’s instructions (Qiagen, Hilden, Germany). Heparinised whole blood was stimulated with antigen coated blood collection tubes (Mitogen, Ag1 [representing CD4 T cell epitopes] and Ag2 [representing CD4 and CD8 T cell epitopes) or unstimulated (Nil) for 16-24 hours. Plasma was collected and stored in -80^o^C until assay. Concentrations of IFN-γ were reported in international units/mL (IU/mL) and background corrected for negative control (Nil).

Supplementary Table 1. Flow cytometry antibody panels and condition for AIM and ICS assays

| Target | Fluorochrome | Clone | Dilution | Company | Catalogue No. |
| --- | --- | --- | --- | --- | --- |
| *AIM – Antibody Panel 1 (room temperature, 20min)* | | | | | |
| PD-1 | BUV615 | EH12 | 1/50 | BD | 612991 |
| CXCR5 | BUV805 | RF8B2 | 1/400 | BD | 741980 |
| CCR4 | BV605 | L291H4 | 1/50 | Biolegend | 359418 |
| CCR6 | BV650 | G034E3 | 1/50 | Biolegend | 353426 |
| CXCR3 | RB780 | 1C6 | 1/50 | BD | 755404 |
| CCR7 | PE | G043H7 | 1/200 | Biolegend | 353204 |
| *AIM – Antibody Panel 2 (4°C, 20min)* | | | | | |
| CD3 | BUV395 | UCHT1 | 1/100 | BD | 563546 |
| CD4 | BUV661 | SK3 | 1/200 | BD | 612962 |
| CD69 | BUV737 | FN50 | 1/200 | BD | 612817 |
| CD154 | BV421 | 24-31 | 1/100 | Biolegend | 310824 |
| Vδ2 | BV480 | B6 | 1/400 | BD | 746567 |
| Vα7.2 | BV711 | 3C10 | 1/50 | Biolegend | 351732 |
| γδTCR | FITC | 11F2 | 1/50 | BD | 347903 |
| CD45RA | PerCPcy5.5 | HI100 | 1/200 | BD | 563429 |
| CD25 | PE-CF594 | M-A251 | 1/50 | BD | 562403 |
| OX-40 | PEcy5 | Ber-ACT35 (ACT35) | 1/100 | Biolegend | 350041 |
| CD161 | PEvio770 | 191B8 | 1/200 | Miltenyi | 130-113-594 |
| CD8 | APC | SK1 | 1/200 | Biolegend | 344722 |
| CD137 | AF647 | 4B4-1 | 1/200 | Biolegend | 309824 |
| CD127 | APC-R700 | HIL-7R-M21 | 1/50 | BD | 565185 |
| Zombie | NIR | - | 1/800 | Biolegend | 423106 |
| *ICS – Antibody Panel 1 (4°C, 20min)* | | | | | |
| CD3 | BUV395 | UCHT1 | 1/100 | BD | 563546 |
| CD4 | BUV661 | SK3 | 1/200 | BD | 612962 |
| Vα7.2 | BV711 | 3C10 | 1/50 | Biolegend | 351732 |
| γδTCR | FITC | 11F2 | 1/50 | BD | 347903 |
| CD45RA | PerCPcy5.5 | HI100 | 1/200 | BD | 563429 |
| Vδ2 | RB780 | B6 | 1/800 | BD | 755725 |
| CD161 | PEvio770 | 191B8 | 1/200 | Miltenyi | 130-113-594 |
| CD8 | APC | SK1 | 1/200 | Biolegend | 344722 |
| Zombie | NIR | - | 1/800 | Biolegend | 423106 |
| *ICS – Antibody Panel 2 (4°C, 20min)* | | | | | |
| IL-2 | BV421 | MQ1-17H12 | 1/100 | Biolegend | 500328 |
| Granzyme b | Pacific Blue | GB11 | 1/100 | Biolegend | 515408 |
| TNF-α | BV785 | MAb11 | 1/100 | Biolegend | 502948 |
| IFN-γ | PE | 4S.B3 | 1/100 | Biolegend | 502509 |
| Perforin | PE-CF594 | δG9 | 1/25 | BD | 563763 |

# Supplementary Results

## Supplementary results QuantiFERON IFN-γ response

For Ag1 (CD4-specific T cell responses) and Ag2 (CD4- and CD8- specific T cell responses), wild-type-specific IFN-γ release at baseline was similar between standard and fractional dose groups, with the exception of the Gam-primed strata had a higher baseline Ag1 IFN-γ level in the fractional dose group (GMR 1.77, 95% CI 0.79 – 4.00) (Figure 3 and Supplementary Table 3). At 28 days post-booster, there was a ~2-fold increase in IFN-γ from baseline for all priming strata. The BBIBP-primed strata had the greatest IFN-γ response to the booster dose, compared to baseline levels for both Ag1 (2.6-3.4-fold increase) and Ag2 (2.5-3.0- fold increase). By 6 months, IFN-γ levels had waned in all groups, although the GMR between standard and fractional doses were maintained for all priming strata (Ag1: GMR 0.96-1.18, Ag2: GMR 0.95-1.34). IFN-γ concentrations at 12 months were similar to 6 months post-vaccination, with similar GMRs between standard and fractional doses for each priming strata (Ag1: 0.56-1.39, Ag2: 0.93-1.33), with the exception of Gam-primed participants which had a lower GMC in the fractional boosted group for Ag1 (GMR: 0.56 95% CI0.31-1.04), yet the 95% CI crossed the null value. There was no JN.1 QuantiFERON kit available at the time of this study.

# Supplementary Tables

Supplementary Table 2. Baseline characteristics of the overall cohort (CMI and main-study) by study group allocation.

|  | All priming strata | | | Primed with ChAd | | Primed with BBIBP | | Primed with Gam | |
| --- | --- | --- | --- | --- | --- | --- | --- | --- | --- |
|  | **Total** | **Standard** | **Fractional** | **Standard** | **Fractional** | **Standard** | **Fractional** | **Standard** | **Fractional** |
|  | **N=598** | **N=299** | **N=299** | **N=65** | **N=64** | **N=200** | **N=199** | **N=34** | **N=36** |
| Participant Age at Enrolment | 44 (32-55) | 44 (32-55) | 44 (33-55) | 34 (32-46) | 40 (34-50) | 48 (31-58) | 48 (32-57) | 43 (32-53) | 41 (35-50) |
| Age group (%) |  |  |  |  |  |  |  |  |  |
| <50 years | 360 (60.2%) | 181 (60.5%) | 179 (59.9%) | 54 (83.1%) | 50 (78.1%) | 103 (51.5%) | 103 (51.8%) | 24 (70.6%) | 26 (72.2%) |
| ≥50 years | 238 (39.8%) | 118 (39.5%) | 120 (40.1%) | 11 (16.9%) | 14 (21.9%) | 97 (48.5%) | 96 (48.2%) | 10 (29.4%) | 10 (27.8%) |
| Participant Sex |  |  |  |  |  |  |  |  |  |
| Male | 273 (45.7%) | 132 (44.1%) | 141 (47.2%) | 32 (49.2%) | 33 (51.6%) | 85 (42.5%) | 86 (43.2%) | 15 (44.1%) | 22 (61.1%) |
| Female | 325 (54.3%) | 167 (55.9%) | 158 (52.8%) | 33 (50.8%) | 31 (48.4%) | 115 (57.5%) | 113 (56.8%) | 19 (55.9%) | 14 (38.9%) |
| BMI, Kg/m^2^ | 25.2  (22.6-28.7) | 25.2  (22.7-28.9) | 25.1  (22.5-28.7) | 26.3  (23.7-30) | 25.4  (23.4-28.4) | 24.6  (22.0-28.7) | 24.8  (22.3-27.9) | 25.3  (24.2-28.7) | 25.8  (23.1-29.8) |
| Days between 1st and 2nd doses | 31 (24–45) | 30 (24–44) | 31 (24–46) | 43 (41-50) | 44 (41-51) | 28 (23-30) | 28 (23-33) | 58 (44-64) | 61 (58-64) |
| Days between 2nd dose and study (3rd) dose | 428  (397–454) | 432  (391–454) | 425  (400–454) | 494  (416-517) | 505  (410-519) | 422  (386-450) | 418  (397-450) | 419  (367-448) | 430  (388-450) |
| Comorbidities |  |  |  |  |  |  |  |  |  |
| Diabetes mellitus | 25 (4.2%) | 17 (5.7%) | 8 (2.7%) | 2 (3.1%) | 1 (1.6%) | 10 (5.0%) | 5 (2.5%) | 5 (14.7%) | 2 (5.6%) |
| Cardiovascular disease | 56 (9.4%) | 26 (8.7%) | 30 (10.0%) | 2 (3.1%) | 5 (7.8%) | 20 (10.0%) | 23 (11.6%) | 4 (11.8%) | 2 (5.6%) |
| Hypertension | 166 (27.8%) | 80 (26.8%) | 86 (28.8%) | 14 (21.5%) | 14 (21.9%) | 59 (29.5%) | 60 (30.2%) | 7 (20.6%) | 12 (33.3%) |
| Chronic kidney disease | 49 (8.2%) | 25 (8.4%) | 24 (8.0%) | 3 (4.6%) | 4 (6.2%) | 18 (9.0%) | 17 (8.5%) | 4 (11.8%) | 3 (8.3%) |
| Cigarette user | 125 (20.9%) | 66 (22.1%) | 59 (19.7%) | 16 (24.6%) | 17 (26.6%) | 37 (18.5%) | 34 (17.1%) | 13 (38.2%) | 8 (22.2%) |
| Currently pregnant | 1 (0.2%) | 1 (0.3%) | 0 (0.0%) | 1 (1.5%) | 0 (0.0%) | 0 (0.0%) | 0 (0.0%) | 0 (0.0%) | 0 (0.0%) |

Supplementary Table 3. Geometric mean concentration anti-spike IgG and geometric mean ratio at day 0, day 28, 6-months and 12-months by priming strata.

|  |  | All priming strata | | ChAd-primed | | BBIBP-primed | | Gam-primed | | |
| --- | --- | --- | --- | --- | --- | --- | --- | --- | --- | --- |
|  |  | **Standard**  **(N=128)** | **Fractional**  **(N=128)** | **Standard**  **(N=50)** | **Fractional**  **(N=51)** | **Standard**  **(N=59)** | **Fractional**  **(N=58)** | **Standard**  **(N=19)** | **Fractional**  **(N=19)** | |
| *Binding Antibodies (IgG) (Wild-type), BAU/mL* | | | | | | | | | |  |
| Day 0 | GMC (IgG)  BAU/ml (95% CI) | 1034  (886 – 1206)  [n=127] | 1054  (915 – 1215)  [n=128] | 1020  (830 – 1254)  [n=50] | 961  (807 – 1145)  [n=51] | 1030  (823 – 1290)  [n=58] | 1107  (866 – 1415)  [n=58] | 1082  (585 – 2002)  [n=19] | 1165  (761 – 1783)  [n=19] | |
|  | GMR (95% CI)  P-value | *Ref* | 1.00  (0.82 – 1.22)  *p*=0.994 | *Ref* | 0.94  (0.72 – 1.22)  *p=*0.633 | *Ref* | 1.06  (0.77 – 1.46)  *p=*0.699 | *Ref* | 0.97  (0.49 – 1.92)  *p=*0.939 | |
| Day 28 | GMC (IgG)  BAU/ml (95% CI) | 5047  (4580 – 5562)  [n=126] | 4559  (4098 – 5071)  [n=124] | 4228  (3653 – 4895)  [n=50] | 3860  (3276 – 4548)  [n=49] | 5759  (4982 – 6658)  [n=58] | 5450  (4639 – 6403)  [n=57] | 5396  (4124 – 7060)  [n=18] | 4074  (3157 – 5257)  [n=18] | |
|  | GMR (95% CI)  P-value | *Ref* | 0.91  (0.80 – 1.03)  *p=*0.155 | *Ref* | 0.90  (0.73 – 1.11)  *p=*0.321 | *Ref* | 0.94  (0.76 – 1.16)  *p=*0.569 | *Ref* | 0.78  (0.54 – 1.11)  *p=*0.163 | |
| 6 months | GMC (IgG)  BAU/ml (95% CI) | 2001  (1775 – 2257)  [n=121] | 1852  (1660 – 2067)  [n=121] | 1618  (1351 – 1938)  [n=46] | 1570  (1316 – 1872)  [n=47] | 2312  (1935 – 2762)  [n=57] | 1944  (1666 -2269)  [n=56] | 2183  (1540 – 3093)  [n=18] | 2456  (1788 – 3372)  [n=18] | |
|  | GMR (95% CI)  P-value | *Ref* | 0.92  (0.79 – 1.07)  *p=*0.293 | *Ref* | 0.97  (0.77 – 1.24)  *p=*0.825 | *Ref* | 0.84  (0.66 – 1.06)  *p=*0.131 | *Ref* | 1.15  (0.73 – 1.81)  *p=*0.529 | |
| 12 months | GMC (IgG)  BAU/ml (95% CI) | 2177  (1920 – 2468)  [n=121] | 2100  (1865 – 2364)  [n=120] | 2247  (1870 – 2698)  [n=46] | 1645  (1390 – 1947)  [n=48] | 2191  (1801 – 2665)  [n=57] | 2284  (1972 – 2645)  [n=54] | 1967  (1327 – 2916)  [n=18] | 3130  (1995 – 4910)  [n=18] | |
|  | GMR (95% CI)  P-value | *Ref* | 0.95  (0.81 – 1.13)  *p=*0.585 | *Ref* | 0.74  (0.58 – 0.94)  *p=*0.014 | *Ref* | 1.04  (0.81 – 1.33)  *p=*0.763 | *Ref* | 1.50  (0.85 – 2.67)  *p=*0.160 | |
| *Binding Antibodies (IgG) (JN.1), RU/mL* | | | | | | | | | |  |
| Day 0 | GMC (IgG)  RU/ml (95% CI) | 59  (48 – 73)  [n=127] | 62  (52 – 74)  [n=126] | 77  (61 – 98)  [n=50] | 60  (49 – 73)  [n=50] | 46  (33 – 65)  [n=58] | 60  (45 – 82)  [n=57] | 62  (28 – 137)  [n=19] | 73  (42 -125)  [n=19] | |
|  | GMR (95% CI)  P-value | *Ref* | 1.02  (0.79 – 1.33)  *p=*0.858 | *Ref* | 0.77  (0.57 – 1.05)  *p=*0.100 | *Ref* | 1.30  (0.85 – 2.01)  *p=*0.226 | *Ref* | 1.00  (0.41 – 2.45)  *p=*0.994 | |
| Day 28 | GMC (IgG)  RU/ml (95% CI) | 462  (399 – 537)  [n=126] | 393  (341 – 454)  [n=122] | 347  (276 – 436)  [n=50] | 293  (240 - 357)  [n=48] | 586  (482 - 711)  [n=58] | 537  (442 – 651)  [n=56] | 482  (287 – 808)  [n=18] | 329  (202 – 536)  [n=18] | |
|  | GMR (95% CI)  P-value | *Ref* | 0.86  (0.70 – 1.04)  *p=*0.115 | *Ref* | 0.88  (0.65 – 1.19)  *p=*0.402 | *Ref* | 0.96  (0.73 – 1.27)  *p=*0.766 | *Ref* | 0.68  (0.35 – 1.30)  *p=*0.231 | |
| 6 months | GMC (IgG)  RU/ml (95% CI) | 134  (115 – 156)  [n=121] | 121  (105 – 140)  [n=119] | 107  (86 – 134)  [n=46] | 98  (79 – 122)  [n=46] | 166  (135 – 205)  [n=57] | 138  (113 – 169)  [n=55] | 120  (68 – 211)  [n=18] | 141  (86 – 231)  [n=18] | |
|  | GMR (95% CI)  P-value | *Ref* | 0.90  (0.74 – 1.10)  *p=*0.318 | *Ref* | 0.98  (0.72 – 1.35)  *p=*0.918 | *Ref* | 0.81  (0.61 – 1.09)  *p=*0.158 | *Ref* | 1.22  (0.60 – 2.49)  *p=*0.568 | |
| 12 months | GMC (IgG)  RU/ml (95% CI) | 114  (98 – 134)  [n=121] | 102  (87 – 119)  [n=118] | 115  (91 – 146)  [n=46] | 73  (59 -91)  [n=47] | 122  (97 – 153)  [n=57] | 124  (102 – 152)  [n=53] | 92  (54 – 159)  [n=18] | 132  (69 – 251)  [n=18] | |
|  | GMR (95% CI)  P-value | *Ref* | 0.89  (0.71 – 1.10)  *p=*0.256 | *Ref* | 0.67  (0.48 – 0.93)  *p=*0.018 | *Ref* | 0.99  (0.73 – 1.35)  *p=*0.969 | *Ref* | 1.24  (0.59 – 2.60)  *p=*0.564 | |

Supplementary Table 4. Median percent inhibition of neutralising antibodies at day 0, day 28, 6-months and 12-months by priming strata.

|  |  | All priming strata | | ChAd-primed | | | BBIBP-primed | | | Gam-primed | | |
| --- | --- | --- | --- | --- | --- | --- | --- | --- | --- | --- | --- | --- |
|  |  | **Standard**  **(N=128)** | **Fractional**  **(N=128)** | **Standard**  **(N=50)** | **Fractional**  **(N=51)** | **Standard**  **(N=59)** | | **Fractional**  **(N=58)** | **Standard**  **(N=19)** | | **Fractional**  **(N=19)** |  |
| *Percent inhibition of neutralising antibodies (Wild-type)* | | | | | | | | | | | | |
| Day 0 | Median inhibition % (IQR) | 81  (77 – 85)  [n=127] | 81  (78 – 84)  [n=128] | 81  (79 – 85)  [n=50] | 80  (77 – 85)  [n=51] | 81  (76 – 85)  [n=58] | | 81  (78 – 84)  [n=58] | 82  (77 – 85)  [n=19] | | 83  (81 – 84)  [n=19] |  |
|  | Mann Whitney U test  P-value | *Ref* | *p=*0.946 | *Ref* | *p=*0.600 | *Ref* | | *p=*0.826 | *Ref* | | *p=*0.885 |  |
| Day 28 | Median inhibition % (IQR) | 81  (78 – 84)  [n=126] | 81  (78 – 84)  [n=124] | 82  (79 – 84)  [n=50] | 81  (77 – 84)  [n=49] | 81  (78 – 84)  [n=58] | | 81  (79 – 84)  [n=57] | 82  (76 – 84)  [n=18] | | 82  (79 – 83)  [n=18] |  |
|  | Mann Whitney U test  P-value | *Ref* | *p=*0.684 | *Ref* | *p=*0.533 | *Ref* | | *p=*0.911 | *Ref* | | *p=*0.864 |  |
| 6 months | Median inhibition % (IQR) | 89  (88 – 91)  [n=121] | 89  (86 – 90)  [n=121] | 89  (88 – 90)  [n=46] | 88  (86 – 90)  [n=47] | 89  (97 – 91)  [n=57] | | 89  (86 – 90)  [n=56] | 90  (88 – 91)  [n=18] | | 89  (88 – 91)  [n=18] |  |
|  | Mann Whitney U test  P-value | *Ref* | *p=*0.073 | *Ref* | *p=*0.085 | *Ref* | | *p=*0.400 | *Ref* | | *p=*0.737 |  |
| 12 months | Median inhibition % (IQR) | 89  (88 – 90)  [n=118] | 89  (87 – 90)  [n=118] | 89  (88 – 90)  [n=46] | 89  (87 – 90)  [n=48] | 89  (87 – 90)  [n=55] | | 89  (88 – 90)  [n=53] | 89  (88 – 90)  [n=17] | | 89  (88 – 89)  [n=17] |  |
|  | Mann Whitney U test  P-value | *Ref* | *p=*0.0.755 | *Ref* | *p=*0.638 | *Ref* | | *p=*0.607 | *Ref* | | *p=*0.265 |  |

Supplementary Table 5. Statistical analysis of T cell memory responses following the AIM assay at day 0, day 28, 6-months and 12-months by priming strata.

|  |  | All priming strata | | ChAd-primed | | | BBIBP-primed | | | Gam-primed | | |
| --- | --- | --- | --- | --- | --- | --- | --- | --- | --- | --- | --- | --- |
|  |  | **Standard**  **(N=128)** | **Fractional**  **(N=128)** | **Standard**  **(N=50)** | **Fractional**  **(N=51)** | **Standard**  **(N=59)** | | **Fractional**  **(N=58)** | **Standard**  **(N=19)** | | **Fractional**  **(N=19)** |  |
| *AIM CD4Mem Total (CD69^+^OX40^+^CD137^+^) Wild-type* | | | | | | | | | | | |  |
| Day 0 | GMC % of CD4Mem  (95% CI) | 0.314  (0.160- 0.615) | 0.183  (0.089 - 0.372) | 0.620  (0.245 - 1.567) | 0.239  (0.079 - 0.723) | 0.148  (0.049 - 0.448) | | 0.124  (0.040 - 0.388) | 0.516  (0.087 - 3.057) | | 0.269  (0.042 - 1.737) |  |
|  | T test  P-value | *Ref* | *p*=0.273 | *Ref* | *p*=0.187 | *Ref* | | *p*=0.830 | *Ref* | | *p*=0.600 |  |
| Day 28 | GMC % of CD4Mem  (95% CI) | 0.688  (0.383 - 1.235) | 0.853  (0.502 - 1.450) | 0.352  (0.121 - 1.027) | 0.631  (0.248 - 1.610) | 1.184  (0.562 - 2.493) | | 0.943  (0.427 - 2.084) | 0.764  (0.149 - 3.919) | | 1.613  (0.866 - 3.004) |  |
|  | T test P-value | *Ref* | *p*=0.594 | *Ref* | *p=*0.414 | *Ref* | | *p*=0.676 | *Ref* | | *p*=0.434 |  |
| 6 months | GMC % of CD4Mem  (95% CI) | 1.164  (0.651 - 2.079) | 2.177  (1.450 - 3.268) | 1.784  (0.848 - 3.754) | 1.703  (0.826 - 3.515) | 1.026  (0.422 - 2.495) | | 2.851  (1.723 - 4.717) | 0.587  (0.067 - 5.118) | | 1.830  (0.471 - 7.108) |  |
|  | T test P-value | *Ref* | *p*=0.084 | *Ref* | *p=*0.928 | *Ref* | | *p*=0.055 | *Ref* | | *p*=0.353 |  |
| 12 months | GMC % of CD4Mem  (95% CI) | 1.048  (0.617 - 1.781) | 0.969  (0.542 - 1.730) | 1.542  (0.765 - 3.107) | 0.448  (0.147 - 1.362) | 0.816  (0.346 - 1.927) | | 1.656  (0.847 - 3.236) | 0.889  (0.174 - 4.530) | | 1.719  (0.424 - 6.971) |  |
|  | T test P-value | *Ref* | *p*=0.842 | *Ref* | *p=*0.063 | *Ref* | | *p*=0.209 | *Ref* | | *p*=0.526 |  |
| *AIM CD4Mem Total (CD69^+^OX40^+^CD137^+^) JN.1* | | | | | | | | | | | |  |
| Day 0 | GMC % of CD4Mem  (95% CI) | 0.105  (0.055 - 0.202) | 0.081  (0.040 - 0.166) | 0.198  (0.071 - 0.550) | 0.105  (0.035 - 0.316) | 0.047  (0.017 - 0.128) | | 0.045  (0.015 - 0.140) | 0.221  (0.041 - 1.187) | | 0.218  (0.035 - 1.357) |  |
|  | T test P-value | *Ref* | *p*=0.596 | *Ref* | *p*=0.398 | *Ref* | | *p*=0.961 | *Ref* | | *p*=0.990 |  |
| Day 28 | GMC % of CD4Mem  (95% CI) | 0.085  (0.042 - 0.174) | 0.094  (0.045 - 0.195) | 0.113  (0.037 - 0.349) | 0.050  (0.015 - 0.169) | 0.053  (0.017 - 0.165) | | 0.120  (0.040 - 0.355) | 0.168  (0.029 - 0.986) | | 0.328  (0.062 - 1.739) |  |
|  | T test P-value | *Ref* | *p*=0.848 | *Ref* | *p*=0.324 | *Ref* | | *p*=0.301 | *Ref* | | *p*=0.576 |  |
| 6 months | GMC % of CD4Mem  (95% CI) | 0.592  (0.334 - 1.049) | 0.821  (0.524 - 1.285) | 0.704  (0.277 - 1.789) | 0.814  (0.375 - 1.764) | 0.358  (0.140 - 0.916) | | 0.899  (0.531 - 1.520) | 2.001  (1.359 - 2.947) | | 0.639  (0.113 - 3.609) |  |
|  | T test P-value | *Ref* | *p*=0.376 | *Ref* | *p*=0.810 | *Ref* | | *p*=0.098 | *Ref* | | *p*=0.182 |  |
| 12 months | GMC % of CD4Mem  (95% CI) | 0.155  (0.077 - 0.312) | 0.135  (0.063 - 0.287) | 0.212  (0.067 - 0.667) | 0.102  (0.029 - 0.355) | 0.111  (0.038 - 0.327) | | 0.138  (0.044 - 0.435) | 0.201  (0.036 - 1.118) | | 0.268  (0.036 - 1.979) |  |
|  | T test P-value | *Ref* | *p*=0.787 | *Ref* | *p*=0.0391 | *Ref* | | *p*=0.784 | *Ref* | | *p*=0.819 |  |
| *AIM CD8Mem Total (CD69^+^CD137^+^) Wild-type* | | | | | | | | | | | |  |
| Day 0 | GMC % of CD8Mem  (95% CI) | 0.006  (0.003 - 0.009) | 0.003  (0.002 - 0.005) | 0.004  (0.002 - 0.010) | 0.004  (0.002 - 0.010) | 0.009  (0.004 - 0.017) | | 0.002  (0.001 - 0.005) | 0.003  (0.001 - 0.012) | | 0.003  (0.001 - 0.013) |  |
|  | T test P-value | *Ref* | *p*=0.111 | *Ref* | *p*=0.986 | *Ref* | | *p*=0.017 | *Ref* | | *p*=0.970 |  |
| Day 28 | GMC % of CD8Mem  (95% CI) | 0.014  (0.008 - 0.023) | 0.009  (0.005 - 0.015) | 0.023  (0.010 - 0.051) | 0.014  (0.006 - 0.030) | 0.011  (0.005 - 0.024) | | 0.006  (0.003 - 0.015) | 0.009  (0.002 - 0.039) | | 0.007  (0.001 - 0.032) |  |
|  | T test P-value | *Ref* | *p*=0.230 | *Ref* | *p*=0.371 | *Ref* | | *p*=0.346 | *Ref* | | *p*=0.804 |  |
| 6 months | GMC % of CD8Mem  (95% CI) | 0.008  (0.005 - 0.014) | 0.017  (0.011 - 0.027) | 0.010  (0.004 - 0.024) | 0.017  (0.010 - 0.030) | 0.008  (0.004 - 0.017) | | 0.013  (0.006 - 0.029) | 0.005  (0.001 - 0.026) | | 0.041  (0.014 - 0.122) |  |
|  | T test P-value | *Ref* | *p*=0.035 | *Ref* | *p*=0.310 | *Ref* | | *p*=0.392 | *Ref* | | *p*=0.032 |  |
| 12 months | GMC % of CD8Mem  (95% CI) | 0.009  (0.006 - 0.014) | 0.005  (0.003 - 0.008) | 0.011  (0.005 - 0.022) | 0.005  (0.002 - 0.011) | 0.006  (0.003 - 0.013) | | 0.003  (0.001 - 0.008) | 0.016  (0.005 - 0.046) | | 0.009  (0.002 - 0.041) |  |
|  | T test P-value | *Ref* | *p*=0.067 | *Ref* | *p*=0.175 | *Ref* | | *p*=0.276 | *Ref* | | *p*=0.544 |  |
| *AIM CD8Mem Total (CD69^+^CD137^+^) JN.1* | | | | | | | | | | | |  |
| Day 0 | GMC % of CD8Mem  (95% CI) | 0.002  (0.001 - 0.003) | 0.002  (0.001 - 0.004) | 0.003  (0.001 - 0.006) | 0.003  (0.001 - 0.007) | 0.001  (0.000 - 0.002) | | 0.002  (0.001 - 0.003) | 0.008  (0.002 - 0.027) | | 0.004  (0.001 - 0.012) |  |
|  | T test P-value | *Ref* | *p*=0.661 | *Ref* | *p*=0.842 | *Ref* | | *p*=0.380 | *Ref* | | *p*=0.354 |  |
| Day 28 | GMC % of CD8Mem  (95% CI) | 0.004  (0.002 - 0.007) | 0.004  (0.002 - 0.006) | 0.005  (0.002 - 0.013) | 0.004  (0.002 - 0.010) | 0.003  (0.001 - 0.007) | | 0.003  (0.001 - 0.006) | 0.003  (0.001 - 0.015) | | 0.007  (0.001 - 0.036) |  |
|  | T test P-value | *Ref* | *p*=0.754 | *Ref* | *p*=0.622 | *Ref* | | *p*=0.732 | *Ref* | | *p*=0.534 |  |
| 6 months | GMC % of CD8Mem  (95% CI) | 0.005  (0.003 - 0.008) | 0.003  (0.002 - 0.005) | 0.008  (0.003 - 0.017) | 0.002  (0.001 - 0.006) | 0.004  (0.002 - 0.008) | | 0.003  (0.001 - 0.007) | 0.002  (0.000 - 0.014) | | 0.005  (0.001 - 0.028) |  |
|  | T test P-value | *Ref* | *p*=0.323 | *Ref* | *p*=0.051 | *Ref* | | *p*=0.859 | *Ref* | | *p*=0.469 |  |
| 12 months | GMC % of CD8Mem  (95% CI) | 0.002  (0.001 - 0.003) | 0.002  (0.001 - 0.003) | 0.003  (0.001 - 0.007) | 0.002  (0.001 - 0.005) | 0.001  (0.000 - 0.002) | | 0.001  (0.001 - 0.003) | 0.001  (0.000 - 0.006) | | 0.002  (0.000 - 0.011) |  |
|  | T test P-value | *Ref* | *p*=0.969 | *Ref* | *p*=0.484 | *Ref* | | *p*=0.756 | *Ref* | | *p*=0.706 |  |

Supplementary Table 6. Statistical analysis of T cell memory responses following the ICS assay at day 0, day 28, 6-months and 12-months by priming strata.

|  |  | All priming strata | | ChAd-primed | | | BBIBP-primed | | | Gam-primed | | |
| --- | --- | --- | --- | --- | --- | --- | --- | --- | --- | --- | --- | --- |
|  |  | **Standard**  **(N=128)** | **Fractional**  **(N=128)** | **Standard**  **(N=50)** | **Fractional**  **(N=51)** | **Standard**  **(N=59)** | | **Fractional**  **(N=58)** | **Standard**  **(N=19)** | | **Fractional**  **(N=19)** |  |
| *ICS CD4Mem Total (IL-2^+^TNF-α^+^IFN-γ^+^) Wild-type* | | | | | | | | | | | |  |
| Day 0 | GMC % of CD4Mem  (95% CI) | 0.007  (0.004 - 0.013) | 0.008  (0.005 - 0.015) | 0.016  (0.007 - 0.038) | 0.015  (0.006 - 0.036) | 0.008  (0.003 - 0.019) | | 0.005  (0.002 - 0.015) | 0.001  (0.000 - 0.004) | | 0.007  (0.001 - 0.032) |  |
|  | T test P-value | *Ref* | *p*=0.700 | *Ref* | *p*=0.921 | *Ref* | | *p*=0.611 | *Ref* | | *p*=0.044 |  |
| Day 28 | GMC % of CD4Mem  (95% CI) | 0.014  (0.008 - 0.026) | 0.015  (0.008 - 0.027) | 0.016  (0.006 - 0.039) | 0.017  (0.007 - 0.042) | 0.018  (0.008 - 0.043) | | 0.025  (0.011 - 0.058) | 0.005  (0.001 - 0.035) | | 0.001  (0.000 - 0.009) |  |
|  | T test P-value | *Ref* | *p*=0.949 | *Ref* | *p*=0.924 | *Ref* | | *p*=0.590 | *Ref* | | *p*=0.282 |  |
| 6 months | GMC % of CD4Mem  (95% CI) | 0.010  (0.006 - 0.019) | 0.011  (0.006 - 0.020) | 0.008  (0.003 - 0.022) | 0.012  (0.005 - 0.031) | 0.017  (0.007 - 0.040) | | 0.013  (0.005 - 0.034) | 0.004  (0.000 -0.027) | | 0.004  (0.001 - 0.022) |  |
|  | T test P-value | *Ref* | *p*=0.933 | *Ref* | *p*=0.496 | *Ref* | | *p*=0.705 | *Ref* | | *p*=0.990 |  |
| 12 months | GMC % of CD4Mem  (95% CI) | 0.019  (0.011 - 0.033) | 0.010  (0.006 - 0.019) | 0.031  (0.013 - 0.073) | 0.007  (0.003 - 0.020) | 0.012  (0.005 - 0.028) | | 0.016  (0.007 - 0.040) | 0.025  (0.007 - 0.089) | | 0.007  (0.001 - 0.036) |  |
|  | T test P-value | *Ref* | *p*=0.126 | *Ref* | *p*=0.027 | *Ref* | | *p*=0.616 | *Ref* | | *p*=0.187 |  |
| *ICS CD4Mem Total (IL-2^+^TNF-α^+^IFN-γ^+^) JN.1* | | | | | | | | | | | |  |
| Day 0 | GMC % of CD4Mem  (95% CI) | 0.005  (0.003 - 0.010) | 0.011  (0.006 - 0.018) | 0.006  (0.002 - 0.014) | 0.012  (0.005 - 0.026) | 0.006  (0.003 - 0.014) | | 0.011  (0.005 - 0.024) | 0.003  (0.001 - 0.018) | | 0.008  (0.001 - 0.045) |  |
|  | T test P-value | *Ref* | *p*=0.085 | *Ref* | *p*=0.263 | *Ref* | | *p*=0.310 | *Ref* | | *p*=0.421 |  |
| Day 28 | GMC % of CD4Mem  (95% CI) | 0.017  (0.010 - 0.028) | 0.007  (0.004 - 0.012) | 0.029  (0.014 - 0.060) | 0.003  (0.001 - 0.008) | 0.011  (0.005 - 0.023) | | 0.013  (0.006 - 0.030) | 0.016  (0.003 - 0.092) | | 0.011  (0.002 - 0.065) |  |
|  | T test P-value | *Ref* | *p*=0.019 | *Ref* | *P*<0.001 | *Ref* | | *p*=0.718 | *Ref* | | *p*=0.756 |  |
| 6 months | GMC % of CD4Mem  (95% CI) | 0.011  (0.007 - 0.019) | 0.030  (0.019 - 0.046) | 0.020  (0.009 - 0.045) | 0.028  (0.014 - 0.055) | 0.007  (0.003 - 0.016) | | 0.036  (0.019 - 0.067) | 0.012  (0.003 - 0.050) | | 0.020  (0.005 - 0.090) |  |
|  | T test P-value | *Ref* | *p*=0.005 | *Ref* | *p*=0.494 | *Ref* | | *p*=0.002 | *Ref* | | *p*=0.591 |  |
| 12 months | GMC % of CD4Mem  (95% CI) | 0.014  (0.008 - 0.024) | 0.010  (0.006 - 0.018) | 0.013  (0.005 - 0.032) | 0.020  (0.010 - 0.041) | 0.014  (0.007 - 0.032) | | 0.004  (0.001 - 0.010) | 0.014  (0.003 - 0.069) | | 0.028  (0.008 - 0.095) |  |
|  | T test P-value | *Ref* | *p*=0.455 | *Ref* | *p*=0.447 | *Ref* | | *p*=0.033 | *Ref* | | *p*=0.463 |  |
| *ICS CD8Mem Total (IL-2^+^TNF-α^+^IFN-γ^+^) Wild-type* | | | | | | | | | | | |  |
| Day 0 | GMC % of CD8Mem  (95% CI) | 0.003  (0.002 - 0.006) | 0.003  (0.001 - 0.005) | 0.004  (0.002 - 0.011) | 0.005  (0.002 - 0.012) | 0.003  (0.001 - 0.008) | | 0.002  (0.001 - 0.005) | 0.002  (0.000 - 0.012) | | 0.001  (0.000 - 0.007) |  |
|  | T test P-value | *Ref* | *p*=0.625 | *Ref* | *p*=0.861 | *Ref* | | *p*=0.515 | *Ref* | | *p*=0.665 |  |
| Day 28 | GMC % of CD8Mem  (95% CI) | 0.003  (0.002 - 0.006) | 0.004  (0.002 - 0.008) | 0.007  (0.003 - 0.020) | 0.004  (0.002 - 0.011) | 0.002  (0.001 - 0.006) | | 0.005  (0.002 - 0.013) | 0.001  (0.000 - 0.008) | | 0.002  (0.000 - 0.014) |  |
|  | T test P-value | *Ref* | *p*=0.659 | *Ref* | *p*=0.475 | *Ref* | | *p*=0.311 | *Ref* | | *p*=0.705 |  |
| 6 months | GMC % of CD8Mem  (95% CI) | 0.004  (0.002 - 0.008) | 0.003  (0.001 - 0.006) | 0.005  (0.002 - 0.013) | 0.002  (0.001 - 0.006) | 0.005  (0.002 - 0.014) | | 0.007  (0.003 - 0.019) | 0.002  (0.000 - 0.012) | | 0.000  (0.000 - 0.002) |  |
|  | T test P-value | *Ref* | *p*=0.393 | *Ref* | *p*=0.344 | *Ref* | | *p*=0.719 | *Ref* | | *p*=0.231 |  |
| 12 months | GMC % of CD8Mem  (95% CI) | 0.005  (0.003 - 0.009) | 0.003  (0.002 - 0.006) | 0.004  (0.002 - 0.011) | 0.003  (0.001 - 0.009) | 0.006  (0.003 - 0.015) | | 0.003  (0.001 - 0.008) | 0.004  (0.001 - 0.026) | | 0.004  (0.001 - 0.027) |  |
|  | T test P-value | *Ref* | *p*=0.312 | *Ref* | *p*=0.732 | *Ref* | | *p*=0.242 | *Ref* | | *p*=0.993 |  |
| *ICS CD8Mem Total (IL-2^+^TNF-α^+^IFN-γ^+^) JN.1* | | | | | | | | | | | |  |
| Day 0 | GMC % of CD8Mem  (95% CI) | 0.003  (0.002 - 0.006) | 0.004  (0.002 - 0.007) | 0.004  (0.002 - 0.011) | 0.004  (0.002 - 0.009) | 0.002  (0.001 - 0.006) | | 0.004  (0.002 - 0.011) | 0.005  (0.001 - 0.028) | | 0.003  (0.001 - 0.016) |  |
|  | T test P-value | *Ref* | *p*=0.709 | *Ref* | *p*=0.879 | *Ref* | | *p*=0.357 | *Ref* | | *p*=0.675 |  |
| Day 28 | GMC % of CD8Mem  (95% CI) | 0.008  (0.005 - 0.015) | 0.008  (0.004 - 0.015) | 0.018  (0.007 - 0.044) | 0.006  (0.002 - 0.016) | 0.005  (0.002 - 0.011) | | 0.012  (0.005 - 0.031) | 0.007  (0.002 - 0.031) | | 0.005  (0.001 - 0.042) |  |
|  | T test P-value | *Ref* | *p*=0.929 | *Ref* | *p*=0.096 | *Ref* | | *p*=0.124 | *Ref* | | *p*=0.807 |  |
| 6 months | GMC % of CD8Mem  (95% CI) | 0.005  (0.003 - 0.009) | 0.008  (0.004 - 0.015) | 0.015  (0.006 - 0.036) | 0.007  (0.003 - 0.018) | 0.003  (0.001 - 0.008) | | 0.011  (0.004 - 0.028) | 0.001  (0.000 - 0.008) | | 0.006  (0.001 - 0.035) |  |
|  | T test P-value | *Ref* | *p*=0.267 | *Ref* | *p*=0.254 | *Ref* | | *p*=0.067 | *Ref* | | *p*=0.218 |  |
| 12 months | GMC % of CD8Mem  (95% CI) | 0.009  (0.005 - 0.017) | 0.006  (0.003 - 0.011) | 0.008  (0.003 - 0.021) | 0.008  (0.003 - 0.022) | 0.011  (0.005 - 0.027) | | 0.003  (0.001 - 0.009) | 0.008  (0.001 - 0.050) | | 0.011  (0.002 - 0.061) |  |
|  | T test P-value | *Ref* | *p*=0.293 | *Ref* | *p*=0.930 | *Ref* | | *p*=0.073 | *Ref* | | *p*=0.812 |  |

Supplementary Table 7. Statistical analysis of the ELISpot assay at day 0, day 28, 6-months and 12-months by priming strata.

|  |  | All priming strata | | ChAd-primed | | BBIBP-primed | | Gam-primed | |
| --- | --- | --- | --- | --- | --- | --- | --- | --- | --- |
|  |  | **Standard**  **(N=96)** | **Fractional**  **(N=96)** | **Standard**  **(N=19)** | **Fractional**  **(N=19)** | **Standard**  **(N=19)** | **Fractional**  **(N=19)** | **Standard**  **(N=19)** | **Fractional**  **(N=19)** |
| *ELISpot Wild-type* | | | | | | | | | |
| Day 0 | IFNγ SFU/10^6^ cells | 43  (24 – 77) | 40  (21 – 75) | 108  (20 - 174) | 85  (53 - 175) | 30  (10 - 58) | 42  (8 - 102) | 128  (52 - 213) | 111  (39 - 178) |
|  | Mann-Whitney  U test  P-value | *Ref* | *p*=0.881 | *Ref* | *p*=0.838 | *Ref* | *p*=0.470 | *Ref* | *p*=0.476 |
| Day 28 | IFNγ SFU/10^6^ cells | 118  (77 – 181) | 127  (87 – 185) | 137  (70 - 380) | 145  (74 - 447) | 62  (43 - 91) | 97  (32 - 196) | 279  (123 - 486) | 171  (44 - 522) |
|  | Mann-Whitney  U test  P-value | *Ref* | *p*=0.914 | *Ref* | *p*=0.963 | *Ref* | *p*=0.482 | *Ref* | *p*=0.475 |
| 6 months | IFNγ SFU/10^6^ cells | 88  (50 – 156) | 80  (43 – 147) | 172  (105 - 370) | 122  (48 – 188) | 89  (51 - 211) | 109  (68 - 204) | 113  (70 - 485) | 177  (75 - 288) |
|  | Mann-Whitney  U test  P-value | *Ref* | *p*=0.741 | *Ref* | *p*=0.240 | *Ref* | *p*=0.635 | *Ref* | *p*=0.823 |
| 12 months | IFNγ SFU/10^6^ cells | 86  (50 – 147) | 82  (48 – 141) | 121  (92 - 227) | 107  (35 - 312) | 80  (57 - 129) | 72  (50 - 123) | 123  (57 - 476) | 198  (93 - 373) |
|  | Mann-Whitney  U test  P-value | *Ref* | *p*=0.939 | *Ref* | *p*=0.725 | *Ref* | *p*=0.508 | *Ref* | *p*=0.865 |
| *ELISpot JN.1* | | | | | | | | | |
| Day 0 | IFNγ SFU/10^6^ cells | 16  (8 – 30) | 29  (15 – 55) | 25  (14 - 97) | 45  (25 - 128) | 7  (0.88 - 36) | 28  (9 - 61) | 38  (18 - 95) | 90  (33 - 145) |
|  | Mann-Whitney  U test  P-value | *Ref* | *p*=0.040 | *Ref* | *p*=0.260 | *Ref* | *p*=0.176 | *Ref* | *p*=0.158 |
| Day 28 | IFNγ SFU/10^6^ cells | 67  (42 – 106) | 92  (61 – 140) | 75  (28 - 247) | 108  (53 - 266) | 53  (33 - 88) | 75  (28 - 138) | 164  (43 - 316) | 118  (48 - 364) |
|  | Mann-Whitney  U test  P-value | *Ref* | *p*=0.389 | *Ref* | *p*=0.448 | *Ref* | *p*=0.318 | *Ref* | *p*=0.890 |
| 6 months | IFNγ SFU/10^6^ cells | 49  (26 – 92) | 81  (49 – 134) | 67  (17 - 255) | 85  (35 - 203) | 77  (31 - 119) | 95  (42 - 269) | 105  (58 - 295) | 152  (50 - 343) |
|  | Mann-Whitney  U test  P-value | *Ref* | *p*=0.231 | *Ref* | *p*=0.716 | *Ref* | *p*=0.100 | *Ref* | *p*=0.892 |
| 12 months | IFNγ SFU/10^6^ cells | 40  (22 – 72) | 62  (40 – 98) | 54  (20 - 143) | 102  (34 - 143) | 60  (12 - 83) | 48  (29 9- 92) | 102  (37 - 269) | 155  (51 - 289) |
|  | Mann-Whitney  U test  P-value | *Ref* | *p*=0.291 | *Ref* | *p*=0.446 | *Ref* | *p*=0.504 | *Ref* | *p*=0.657 |

Supplementary Table 8 Statistical analysis of the multiplex cytokine assays at day 0, day 28, 6-months and 12-months by priming strata.

|  |  | All priming strata | | ChAd-primed | | BBIBP-primed | | Gam-primed | |
| --- | --- | --- | --- | --- | --- | --- | --- | --- | --- |
|  |  | **Standard**  **(N=128)** | **Fractional**  **(N=128)** | **Standard**  **(N=50)** | **Fractional**  **(N=51)** | **Standard**  **(N=59)** | **Fractional**  **(N=58)** | **Standard**  **(N=19)** | **Fractional**  **(N=19)** |
| *Wild-type multiplex cytokines* | | | | | | | | | |
| *IL-2* | | | | | | | | | |
| Day 0 | GMC  (95% CI) | 2.77  (1.54 - 4.98) | 3.64  (2.15 - 6.16) | 4.17  (1.60 - 10.90) | 2.62  (1.03 - 6.66) | 2.32  (0.98 - 5.49) | 3.04  (1.43 - 6.46) | 14.36  (4.98 - 41.41) | 1.63  (0.30 - 8.80) |
|  | T test  P-value | *Ref* | *p*=0.326 | *Ref* | *p*=0.486 | *Ref* | *p*=0.637 | *Ref* | *p*=0.028 |
| Day 28 | GMC  (95% CI) | 11.71  (7.45 - 18.39) | 14.74  (9.30 - 23.35) | 6.97  (2.98 - 16.29) | 11.80  (5.43 - 25.63) | 15.63  (8.46 - 28.88) | 17.97  (9.46 - 34.15) | 14.57  (3.50 - 60.61) | 21.28  (12.62 - 35.87) |
|  | T test  P-value | *Ref* | *p*=0.327 | *Ref* | *p*=0.360 | *Ref* | *p*=0.754 | *Ref* | *p*=628 |
| 6 months | GMC  (95% CI) | 4.38  (2.30 - 8.35) | 6.22  (3.43 - 11.29) | 1.48  (0.42 - 5.17) | 4.21  (1.42 - 12.44) | 10.10  (4.66 - 21.87) | 9.24  (4.25 - 20.06) | 5.06  (0.88 - 29.28) | 4.08  (0.71 - 23.63) |
|  | T test  P-value | *Ref* | *p*=0.523 | *Ref* | *p*=0.205 | *Ref* | *p*=0.871 | *Ref* | *p*=0.856 |
| 12 months | GMC  (95% CI) | 7.61  (4.40 - 13.16) | 10.71  (6.69 - 17.14) | 12.55  (5.60 - 28.11) | 5.91  (2.47 - 14.11) | 5.39  (2.28 - 12.77) | 16.04  (8.67 - 29.68) | 16.14  (5.54 - 47.07) | 6.72  (1.45 - 31.13) |
|  | T test  P-value | *Ref* | *p*=0.846 | *Ref* | *p*=0.206 | *Ref* | *p*=0.045 | *Ref* | *p*=0.330 |
| *IL-4* | | | | | | | | | |
| Day 0 | GMC  (95% CI) | 0.31  (0.20 - 0.47) | 0.23  (0.15 - 0.35) | 0.31  (0.16 - 0.62) | 0.23  (0.12 - 0.46) | 0.22  (0.12 - 0.43) | 0.24  (0.13 - 0.42) | 0.78  (0.29 - 2.11) | 0.21  (0.06 - 0.72) |
|  | T test  P-value | *Ref* | *p*=0.332 | *Ref* | *p*=0.538 | *Ref* | *p*=0.877 | *Ref* | *p*=0.086 |
| Day 28 | GMC  (95% CI) | 0.62  (0.41 - 0.93) | 0.80  (0.54 - 1.17) | 0.42  (0.21 - 0.84) | 0.69  (0.36 - 1.35) | 0.73  (0.39 - 1.34) | 0.95  (0.56 - 1.61) | 1.03  (0.37 - 2.84) | 0.66  (0.21 - 2.06) |
|  | T test  P-value | *Ref* | *p*=0.364 | *Ref* | *p*=0.297 | *Ref* | *p*=0.514 | *Ref* | *p*=0.543 |
| 6 months | GMC  (95% CI) | 0.39  (0.24 - 0.62) | 0.52  (0.33 - 0.82) | 0.32  (0.14 - 0.72) | 0.52  (0.24 - 1.13) | 0.51  (0.27 - 0.98) | 0.62  (0.34 - 1.15) | 0.25  (0.06 - 0.97) | 0.32  (0.08 - 1.21) |
|  | T test  P-value | *Ref* | *p*=0.356 | *Ref* | *p*=0.385 | *Ref* | *p*=0.653 | *Ref* | *p*=0.785 |
| 12 months | GMC  (95% CI) | 0.56  (0.36 - 0.87) | 0.60  (0.39 - 0.91) | 0.71  (0.34 - 1.48) | 0.45  (0.22 - 0.93) | 0.45  (0.24 - 0.85) | 0.66  (0.35 - 1.26) | 0.65  (0.19 - 2.28) | 0.90  (0.30 - 2.66) |
|  | T test  P-value | *Ref* | *p*=0.854 | *Ref* | *p*=0.385 | *Ref* | *p*=0.390 | *Ref* | *p*=0.691 |
| *IL-6* |  |  |  |  |  |  |  |  |  |
| Day 0 | GMC  (95% CI) | 27.21  (12.16 - 60.87) | 13.88  (6.02 - 32.00) | 19.32  (5.20 - 71.83) | 12.72  (3.08 - 52.58) | 21.40  (6.26 - 73.16) | 24.56  (8.08 - 74.65) | 135.15  (18.74 - 974.61) | 3.34  (0.24 - 45.49) |
|  | T test  P-value | *Ref* | *p*=0.252 | *Ref* | *p*=0.665 | *Ref* | *p*=0.869 | *Ref* | *p*=0.023 |
| Day 28 | GMC  (95% CI) | 38.29  (16.51 - 88.81) | 80.51  (37.63 - 172.23) | 12.56  (3.13 - 50.34) | 70.30  (19.88 - 248.62) | 100.15  (31.01- 323.38) | 91.64  (28.44 - 295.26) | 37.95  (3.14 - 458.34) | 77.27  (14.02 - 425.91) |
|  | T test  P-value | *Ref* | *p*=0.197 | *Ref* | *p*=0.068 | *Ref* | *p*=0.915 | *Ref* | *p*=0.636 |
| 6 months | GMC  (95% CI) | 15.89  (6.11 - 41.34) | 25.05  (9.97 - 62.96) | 9.52  (1.70 - 53.17) | 19.96  (4.18 - 95.39) | 33.08  (9.51 - 115.14) | 39.16  (10.54 - 145.46) | 4.69  (0.22 - 99.25) | 11.70  (0.80 - 170.44) |
|  | T test  P-value | *Ref* | *p*=0.498 | *Ref* | *p*=0.521 | *Ref* | *p*=0.852 | *Ref* | *p*=0.635 |
| 12 months | GMC  (95% CI) | 43.84  (18.40 - 104.46) | 42.07  (17.39 - 101.80) | 39.19  (8.22 - 186.95) | 23.59  (5.44 - 102.28) | 54.92  (17.20 - 175.37) | 60.42  (15.92 - 229.32) | 28.41  (1.99 - 405.37) | 68.33  (7.04 - 663.63) |
|  | T test  P-value | *Ref* | *p*=0.948 | *Ref* | *p*=0.634 | *Ref* | *p*=0.914 | *Ref* | *p*=0.600 |
| *IFN-γ* |  |  |  |  |  |  |  |  |  |
| Day 0 | GMC  (95% CI) | 1.79  (1.07 - 2.99) | 1.52  (0.91 - 2.55) | 2.55  (1.12 - 5.83) | 2.02  (0.89 - 4.56) | 0.82  (0.38 - 1.77) | 1.04  (0.50 - 2.14) | 7.44  (2.54 - 21.81) | 2.21  (0.39 - 12.61) |
|  | T test  P-value | *Ref* | *p*=0.654 | *Ref* | *p*=0.686 | *Ref* | *p*=0.661 | *Ref* | *p*=0.221 |
| Day 28 | GMC  (95% CI) | 7.36  (4.86 - 11.14) | 10.02  (7.16 - 14.03) | 5.69  (2.55 - 12.70) | 10.01  (5.95 - 16.85) | 7.12  (4.40 - 11.52) | 9.69  (6.39 - 14.68) | 16.47  (5.08 - 53.43) | 11.41  (2.28 - 57.15) |
|  | T test  P-value | *Ref* | *p*=0.256 | *Ref* | *p*=0.240 | *Ref* | *p*=0.335 | *Ref* | *p*=0.692 |
| 6 months | GMC  (95% CI) | 4.69  (2.57 - 8.57) | 5.48  (3.06 - 9.81) | 3.62  (1.26 - 10.36) | 5.25  (1.83 - 15.04) | 7.25  (3.15 - 16.70) | 5.28  (2.29 - 12.16) | 2.02  (0.35 - 11.80) | 6.87  (1.73 - 27.18) |
|  | T test  P-value | *Ref* | *p*=0.713 | *Ref* | *p*=0.617 | *Ref* | *p*=0.591 | *Ref* | *p*=0.250 |
| 12 months | GMC  (95% CI) | 4.92  (2.96 - 8.18) | 7.44  (4.78 - 11.57) | 7.26  (3.10 - 16.99) | 5.83  (2.65 - 12.81) | 2.88  (1.32 - 6.27) | 7.68  (4.14 - 14.22) | 10.23  (3.53 - 29.66) | 12.83  (4.24 - 38.85) |
|  | T test  P-value | *Ref* | *p*=0.226 | *Ref* | *p*=0.703 | *Ref* | *p*=0.053 | *Ref* | *p*=0.758 |
| *TNF-α* |  |  |  |  |  |  |  |  |  |
| Day 0 | GMC  (95% CI) | 14.22  (6.56 - 30.83) | 5.20  (2.36 - 11.44) | 11.97  (3.09 - 46.42) | 5.94  (1.60 - 22.07) | 8.99  (2.79 - 28.93) | 6.16  (2.01 - 18.90) | 87.86  (23.19 - 332.79) | 2.26  (0.21 - 23.98) |
|  | T test  P-value | *Ref* | *p*=0.073 | *Ref* | *p*=0.457 | *Ref* | *p*=0.642 | *Ref* | *p*=0.007 |
| Day 28 | GMC  (95% CI) | 40.64  (19.21 - 85.98) | 78.18  (41.08 - 148.80) | 16.94  (4.36 - 65.88) | 83.23  (29.98 - 231.10) | 66.23  (24.13 - 181.78) | 82.84  (31.56 - 217.42) | 93.70  (14.57 - 602.44) | 51.78  (6.90 - 388.74) |
|  | T test  P-value | *Ref* | *p*=0.193 | *Ref* | *p*=0.063 | *Ref* | *p*=0.749 | *Ref* | *p*=0.650 |
| 6 months | GMC  (95% CI) | 10.74  (4.24 - 27.18) | 9.85  (3.86 - 25.13) | 3.95  (0.71 - 22.11) | 6.24  (1.19 - 32.79) | 33.05  (10.40 - 105.01) | 17.79  (4.95 - 64.00) | 2.91  (0.18 - 47.28) | 5.38  (0.38 - 75.62) |
|  | T test  P-value | *Ref* | *p*=0.897 | *Ref* | *p*=0.701 | *Ref* | *p*=0.473 | *Ref* | *p*=0.736 |
| 12 months | GMC  (95% CI) | 17.26  (7.12 - 41.84) | 32.22  (14.46 - 71.80) | 33.63  (7.90 - 143.17) | 17.45  (4.51 - 67.61) | 9.02  (2.38 - 34.18) | 47.17  (14.33 - 155.34) | 26.47  (2.57 - 272.31) | 54.24  (7.30 - 403.08) |
|  | T test  P-value | *Ref* | *p*=0.302 | *Ref* | *p*=0.507 | *Ref* | *p*=0.068 | *Ref* | *p*=0.626 |
| *JN.1 multiplex cytokines* | | | | | | | | | |
| *IL-2* |  |  |  |  |  |  |  |  |  |
| Day 0 | GMC  (95% CI) | 0.79  (0.43 - 1.47) | 0.65  (0.35 - 1.19) | 1.76  (0.65 - 4.74) | 0.71  (0.26 - 1.92) | 0.39  (0.16 - 0.92) | 0.56  (0.23 - 1.35) | 0.94  (0.12 - 7.39) | 0.83  (0.12 - 5.73) |
|  | T test  P-value | *Ref* | *p*=0.636 | *Ref* | *p*=0.197 | *Ref* | *p*=0.562 | *Ref* | *p*=0.923 |
| Day 28 | GMC  (95% CI) | 1.77  (0.91 - 3.47) | 2.75  (1.43 - 5.28) | 1.50  (0.50 - 4.50) | 3.52  (1.28 - 9.69) | 1.53  (0.56 - 4.18) | 2.14  (0.79 - 5.79) | 5.53  (0.87 - 35.26) | 3.27  (0.39 - 27.40) |
|  | T test  P-value | *Ref* | *p*=0.356 | *Ref* | *p*=0.255 | *Ref* | *p*=0.638 | *Ref* | *p*=0685 |
| 6 months | GMC  (95% CI) | 0.93  (0.47 - 1.83) | 1.45  (0.78 - 2.70) | 1.23  (0.39 - 3.84) | 1.02  (0.35 - 2.97) | 0.89  (0.34 - 2.33) | 2.31  (1.02 - 5.26) | 0.40  (0.04 - 4.27) | 0.71  (0.08 - 6.42) |
|  | T test  P-value | *Ref* | *p*=0.337 | *Ref* | *p*=0.808 | *Ref* | *p*=0.134 | *Ref* | *p*=0.695 |
| 12 months | GMC  (95% CI) | 0.90  (0.42 - 1.93) | 2.09  (1.00 - 4.33) | 0.94  (0.27 - 3.34) | 2.12  (0.67 - 6.72) | 0.78  (0.26 - 2.36) | 1.84  (0.60 - 5.62) | 1.47  (0.13 - 16.71) | 3.20  (0.33 - 31.17) |
|  | T test  P-value | *Ref* | *p*=0.117 | *Ref* | *p*=0.339 | *Ref* | *p*=0.276 | *Ref* | *p*=0.613 |
| *IL-4* |  |  |  |  |  |  |  |  |  |
| Day 0 | GMC  (95% CI) | 0.09  (0.06 - 0.15) | 0.06  (0.04 - 0.10) | 0.13  (0.06 - 0.28) | 0.07  (0.04 - 0.15) | 0.08  (0.04 - 0.14) | 0.05  (0.03 - 0.09) | 0.07  (0.01 - 0.34) | 0.11  (0.03 - 0.43) |
|  | T test  P-value | *Ref* | *p*=0.205 | *Ref* | *p*=0.291 | *Ref* | *p*=0.247 | *Ref* | *p*=0.643 |
| Day 28 | GMC  (95% CI) | 0.13  (0.08 - 0.21) | 0.20  (0.12 - 0.34) | 0.12  (0.06 - 0.28) | 0.28  (0.13 - 0.65) | 0.10  (0.05 - 0.21) | 0.13  (0.06 - 0.29) | 0.36  (0.08 - 1.67) | 0.38  (0.08 - 1.73) |
|  | T test  P-value | *Ref* | *p*=0.212 | *Ref* | *p*=0.153 | *Ref* | *p*=0.626 | *Ref* | *p*=0.964 |
| 6 months | GMC  (95% CI) | 0.07  (0.04 - 0.11) | 0.10  (0.06 - 0.15) | 0.08  (0.04 - 0.17) | 0.13  (0.06 - 0.27) | 0.06  (0.03 - 0.12) | 0.08  (0.04 - 0.15) | 0.07  (0.01 - 0.36) | 0.08  (0.02 - 0.33) |
|  | T test  P-value | *Ref* | *p*=0.292 | *Ref* | *p*=0.330 | *Ref* | *p*=0.591 | *Ref* | *p*=0.924 |
| 12 months | GMC  (95% CI) | 0.14  (0.08 - 0.24) | 0.21  (0.12 - 0.36) | 0.14  (0.06 - 0.34) | 0.18  (0.07 - 0.44) | 0.15  (0.07 - 0.32) | 0.20  (0.09 - 0.45) | 0.11  (0.02 - 0.60) | 0.38  (0.06 - 2.29) |
|  | T test  P-value | *Ref* | *p*=0.303 | *Ref* | *p*=0.680 | *Ref* | *p*=0.576 | *Ref* | *p*=0.279 |
| *IL-6* |  |  |  |  |  |  |  |  |  |
| Day 0 | GMC  (95% CI) | 1.51  (0.60 - 3.79) | 0.68  (0.28 - 1.67) | 3.73  (0.81 - 17.20) | 0.87  (0.20 - 3.75) | 0.80  (0.23 - 2.80) | 0.39  (0.11 - 1.44) | 0.92  (0.04 - 22.73) | 2.52  (0.14 - 44.12) |
|  | T test  P-value | *Ref* | *p*=0.220 | *Ref* | *p*= 0.168 | *Ref* | *p*= 0.431 | *Ref* | *p*= 0.613 |
| Day 28 | GMC  (95% CI) | 1.22  (0.43 - 3.48) | 3.17  (1.07 - 9.36) | 0.72  (0.13 - 3.87) | 8.08  (1.46 - 44.71) | 1.45  (0.31 - 6.75) | 1.49  (0.31 - 7.25) | 3.50  (0.10 - 121.35) | 2.51  (0.06 - 108.81) |
|  | T test  P-value | *Ref* | *p*=0.210 | *Ref* | *p*= 0.045 | *Ref* | *p*= 0.982 | *Ref* | *p*= 0.889 |
| 6 months | GMC  (95% CI) | 0.85  (0.32 - 2.23) | 1.04  (0.40 - 2.69) | 1.28  (0.27 - 6.01) | 2.06  (0.41 - 10.22) | 0.94  (0.23 - 3.79) | 0.72  (0.19 - 2.71) | 0.10  (0.00 - 3.30) | 0.47  (0.02 - 10.97) |
|  | T test  P-value | *Ref* | *p*=0.760 | *Ref* | *p*= 0.669 | *Ref* | *p*= 0.777 | *Ref* | *p*= 0.474 |
| 12 months | GMC  (95% CI) | 2.00  (0.65 - 6.13) | 3.43  (1.10 - 10.63) | 2.54  (0.41 - 15.74) | 4.31  (0.74 - 25.04) | 1.72  (0.33 - 9.01) | 2.24  (0.41 - 12.27) | 1.77  (0.05 - 63.23) | 7.82  (0.15 - 399.68) |
|  | T test  P-value | *Ref* | *p*=0.503 | *Ref* | *p*= 0.675 | *Ref* | *p*= 0.824 | *Ref* | *p*= 0545 |
| *IFN-γ* |  |  |  |  |  |  |  |  |  |
| Day 0 | GMC  (95% CI) | 0.44  (0.25 - 0.78) | 0.42  (0.25 - 0.71) | 1.16  (0.48 - 2.76) | 0.70  (0.30 - 1.62) | 0.16  (0.08 - 0.35) | 0.24  (0.11 - 0.51) | 0.86  (0.11 - 6.82) | 0.66  (0.13 - 3.38) |
|  | T test  P-value | *Ref* | *p*=0.891 | *Ref* | *p*=0.401 | *Ref* | *p*=0.507 | *Ref* | *p*=0.829 |
| Day 28 | GMC  (95% CI) | 1.13  (0.62 - 2.09) | 1.59  (0.89 - 2.86) | 1.47  (0.55 - 3.89) | 2.29  (0.96 - 5.47) | 0.79  (0.34 - 1.85) | 1.06  (0.44 - 2.55) | 1.98  (0.18 - 21.90) | 2.49  (0.28 - 22.09) |
|  | T test  P-value | *Ref* | *p*=0.426 | *Ref* | *p*=0.495 | *Ref* | *p*=0.638 | *Ref* | *p*=0.879 |
| 6 months | GMC  (95% CI) | 0.93  (0.48 - 1.82) | 1.20  (0.65 - 2.20) | 2.16  (0.77 - 6.09) | 3.10  (1.22 - 7.90) | 0.43  (0.17 - 1.11) | 0.59  (0.25 - 1.42) | 1.92  (0.17 - 21.33) | 0.82  (0.10 - 6.69) |
|  | T test  P-value | *Ref* | *p*=0.588 | *Ref* | *p*=0.601 | *Ref* | *p*=0.634 | *Ref* | *p*=0.559 |
| 12 months | GMC  (95% CI) | 0.66  (0.34 - 1.29) | 1.19  (0.61 - 2.35) | 0.83  (0.29 - 2.41) | 0.87  (0.30 - 2.58) | 0.67  (0.25 - 1.79) | 1.32  (0.48 - 3.62) | 0.30  (0.03 - 3.01) | 2.43  (0.27 - 21.60) |
|  | T test  P-value | *Ref* | *p*=0.217 | *Ref* | *p*=0.945 | *Ref* | *p*=0.336 | *Ref* | *p*=0.160 |
| *TNF-α* |  |  |  |  |  |  |  |  |  |
| Day 0 | GMC  (95% CI) | 1.41  (0.60 - 3.31) | 0.58  (0.24 - 1.36) | 2.99  (0.74 - 12.15) | 1.03  (0.25 - 4.18) | 0.67  (0.20 - 2.25) | 0.34  (0.10 - 1.18) | 2.14  (0.14 - 33.99) | 0.60  (0.04 - 9.96) |
|  | T test  P-value | *Ref* | *p*=0.146 | *Ref* | *p*=0.281 | *Ref* | *p*=0.442 | *Ref* | *p*=0.487 |
| Day 28 | GMC  (95% CI) | 2.54  (0.94 - 6.84) | 6.00  (2.19 - 16.40) | 3.17  (0.64 - 15.77) | 9.59  (1.96 - 47.01) | 1.30  (0.30 - 5.65) | 4.08  (0.92 - 18.07) | 17.56  (1.10 - 279.55) | 5.52  (0.16 - 188.40) |
|  | T test  P-value | *Ref* | *p*=0.229 | *Ref* | *p*=0.326 | *Ref* | *p*=0.274 | *Ref* | *p*=0.570 |
| 6 months | GMC  (95% CI) | 0.91  (0.36 - 2.30) | 2.32  (0.96 - 5.58) | 1.68  (0.36 - 7.77) | 3.23  (0.75 - 13.95) | 0.86  (0.23 - 3.23) | 2.17  (0.61 - 7.75) | 0.12  (0.01 - 2.21) | 0.96  (0.06 - 15.55) |
|  | T test  P-value | *Ref* | *p*=0.147 | *Ref* | *p*=0.533 | *Ref* | *p*=0.311 | *Ref* | *p*=0.263 |
| 12 months | GMC  (95% CI) | 2.24  (0.78 - 6.41) | 5.31  (1.88 - 15.03) | 2.65  (0.47 - 14.94) | 4.00  (0.74 - 21.75) | 1.50  (0.32 - 7.03) | 5.40  (1.13 - 25.93) | 7.18  (0.29 - 179.92) | 13.37  (0.67 - 266.67) |
|  | T test  P-value | *Ref* | *p*=0.249 | *Ref* | *p*=0.732 | *Ref* | *p*=0.246 | *Ref* | *p*=0.759 |

## Supplementary Figures

**
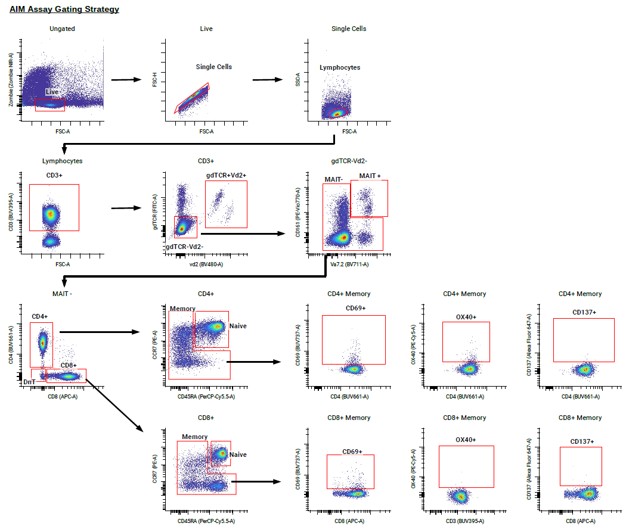

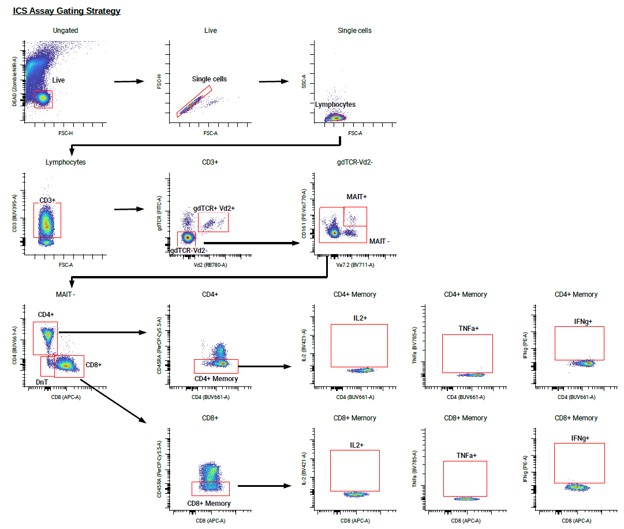
**

**Supplementary Figure 1. Gating strategy for the AIM and ICS assay.**

**
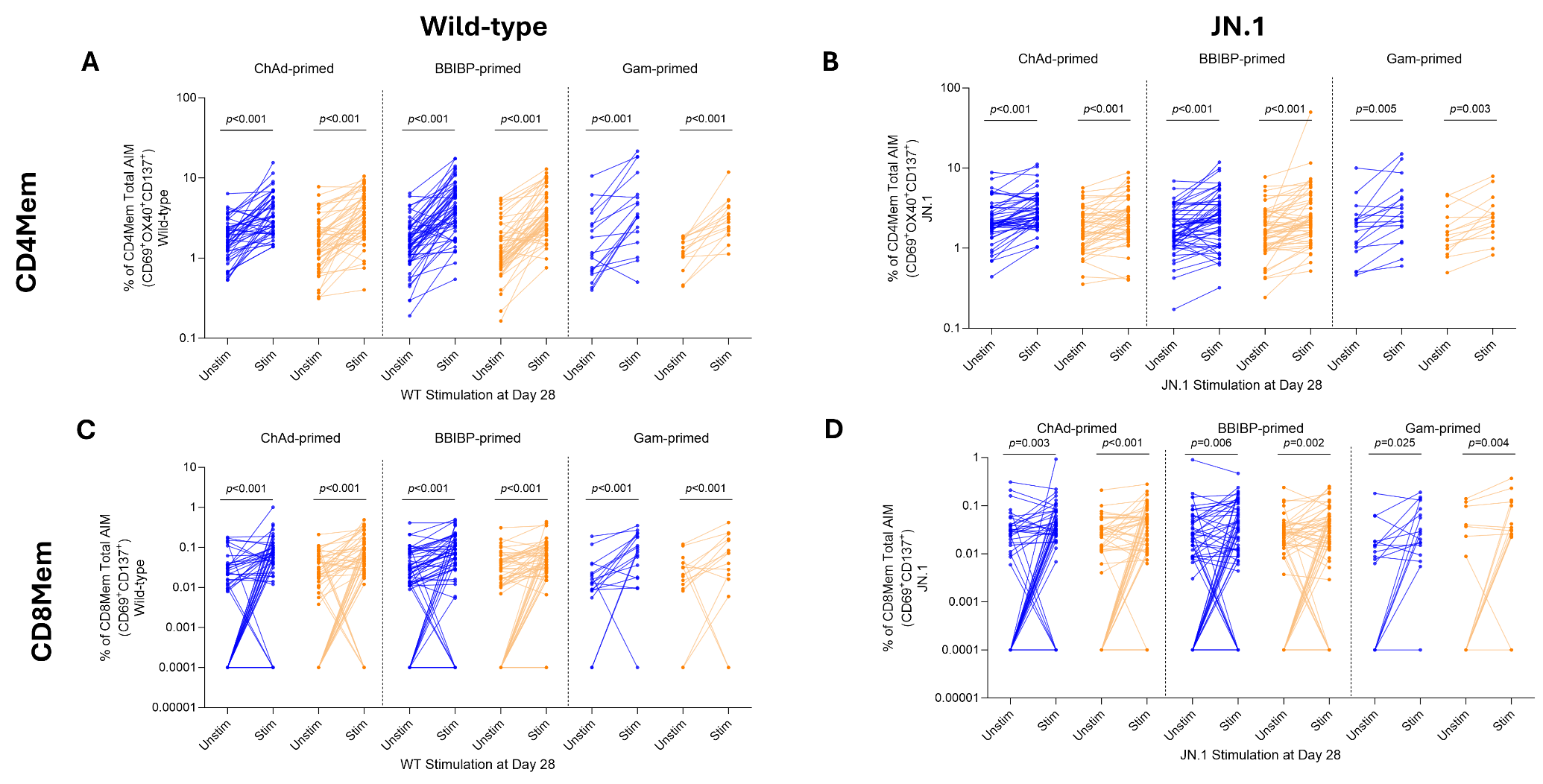
Supplementary Figure 2. T cell memory stimulatory responses by AIM assay at day 28.** Total stimulatory responses CD4mem AIM (CD69^+^, OX40^+^ or CD137^+^) for wild-type (A) and JN.1 (B). Total CD8mem AIM (CD69^+^CD137^+^) for wild-type (C) and JN.1 (D). Results displayed are unstimulated (media) and paired wild-type peptide stimulatory response, and unstimulated (DMSO) responses with paired stimulatory JN.1 peptide responses at day 28, with results shown as GMC ± 95% CI. A paired Wilcoxon signed-rank test was done comparing day 28 post vaccination responses between unstimulated and stimulated conditions.

**
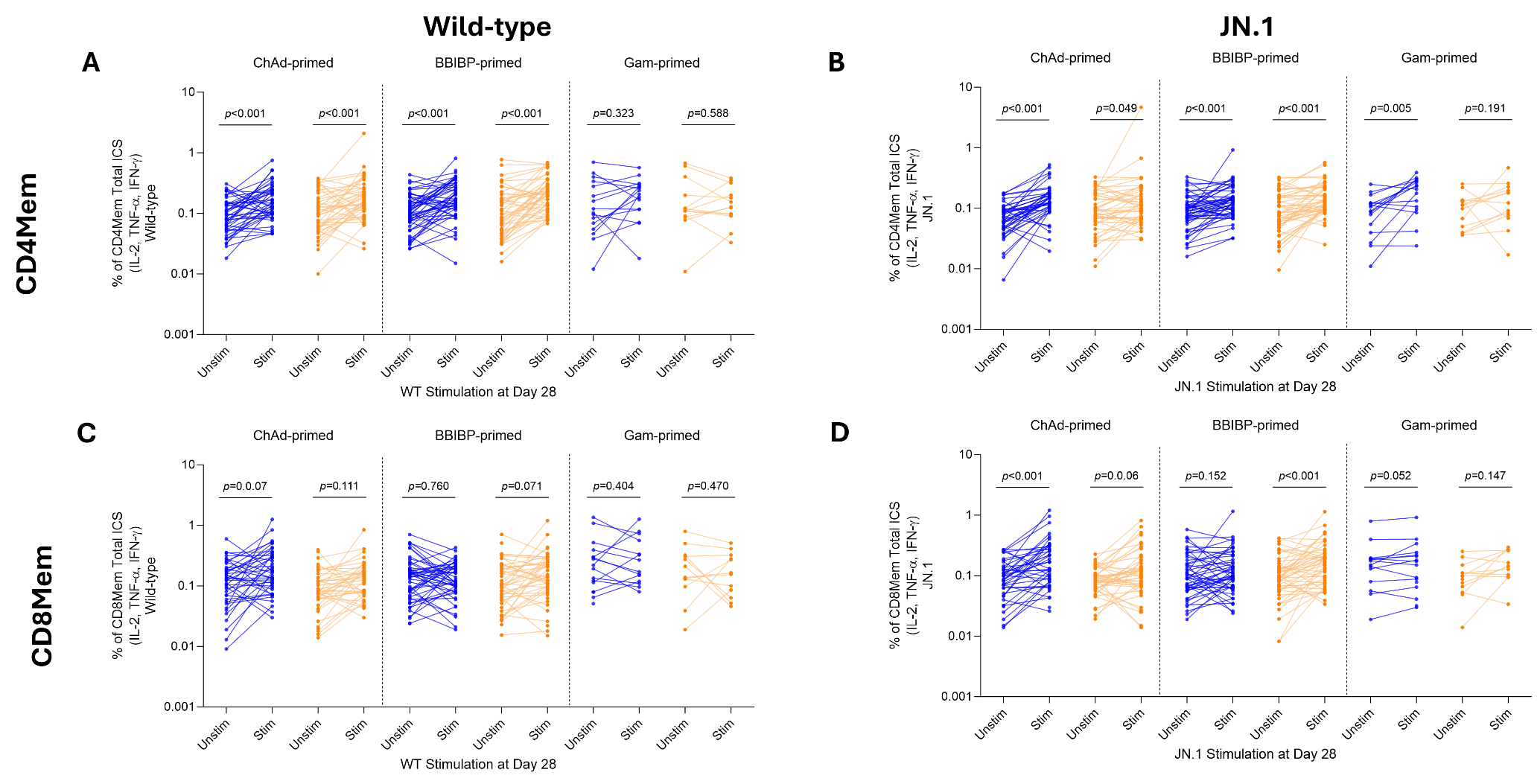
Supplementary Figure 3. T cell memory stimulatory responses by ICS assay at day 28.** Total stimulatory responses CD4mem ICS (IL-2^+^, TNF-α^+^ or IFN-γ^+^) for wild-type (A) and JN.1 (B). Total CD8mem ICS (IL-2^+^, TNF-α^+^ or IFN-γ^+^) for wild-type (C) and JN.1 (D). Results displayed are unstimulated (media) and paired wild-type peptide stimulatory response, and unstimulated (DMSO) responses with paired stimulatory JN.1 peptide responses at day 28, with results shown as GMC ± 95% CI. A paired Wilcoxon signed-rank test was done comparing day 28 post vaccination responses between unstimulated and stimulated conditions.

***
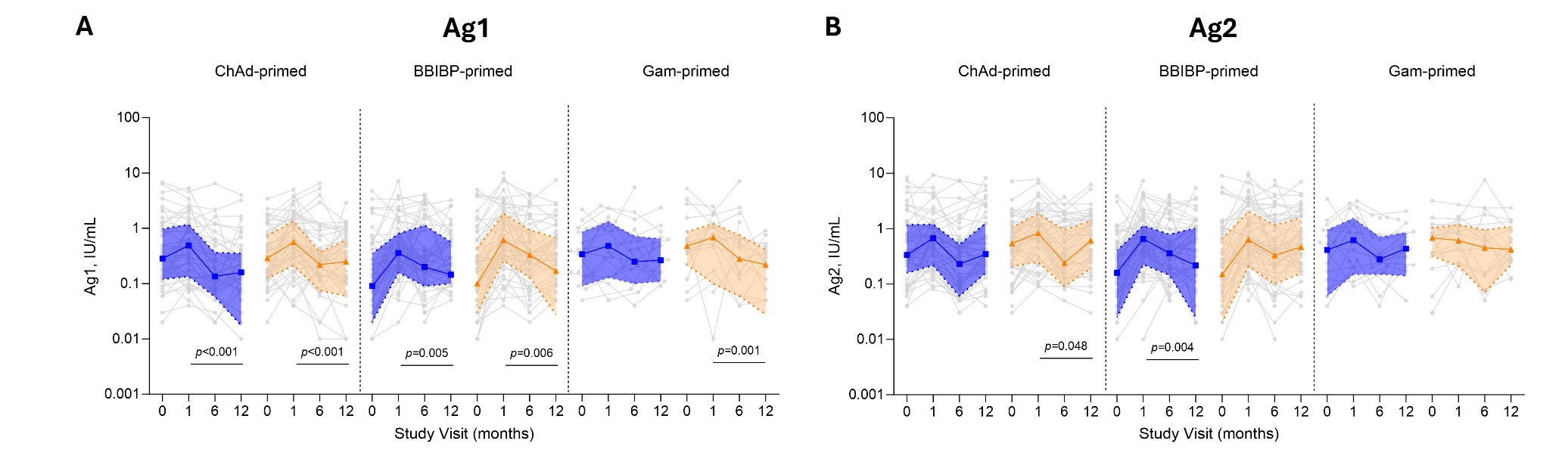
*Supplementary Figure 4. IFN-γ response by QuantiFERON assay up to 12 months following fractional (orange) or standard (blue) dose booster vaccination.** IFN-γ release following whole blood stimulation with wild-type (Wuhan-Hu-1) spike protein antigen 1 (Ag1, A) and antigen 2 (Ag2, B). Results are calculated as antigen (Ag1 or Ag2) minus baseline (Nil) and displayed with median ± IQR, with a Mann-Whitney U test performed between fractional dose/standard dose at all timepoints, and with a *p*-value for paired Wilcoxon signed-rank test between day 28 and 12-months post-vaccination. IU: International Units.


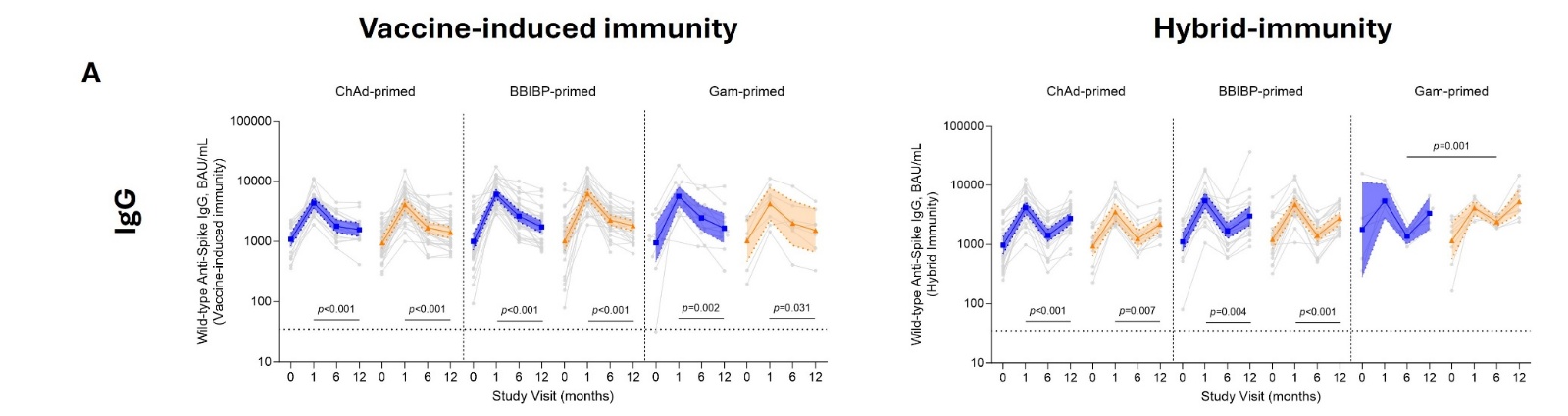

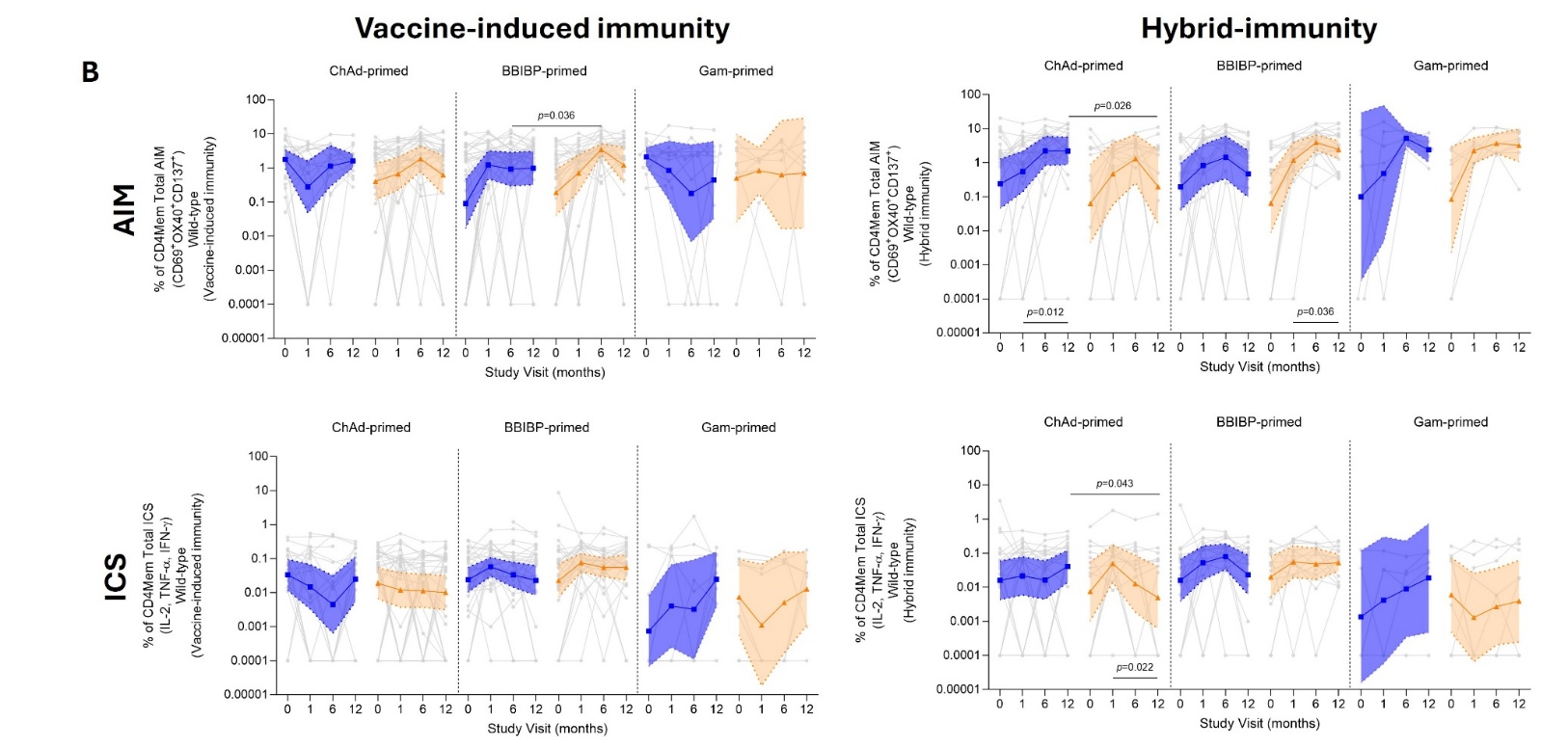


**Supplementary Figure 5*.* Vaccine-induced immunity (blue) and hybrid-immunity (pink) responses following fractional or standard dose booster vaccination against wild-type strain.**  Vaccine-induced immunity and hybrid-immunity between 6-12 months post-vaccine was evaluated for (A) IgG and (B) CD4Mem AIM (CD60+, OX40+, CD137+) and ICS (IL-2+, TNF-α+, IFN-γ+) responses against wild-type strain. Responses over the 12-month period are presented as GMC ± 95% CI, with data log transformed for a parametric t-test performed between standard and fractional dose groups, with a paired Wilcoxon signed-rank test comparing day 28 and 12-months post vaccination.


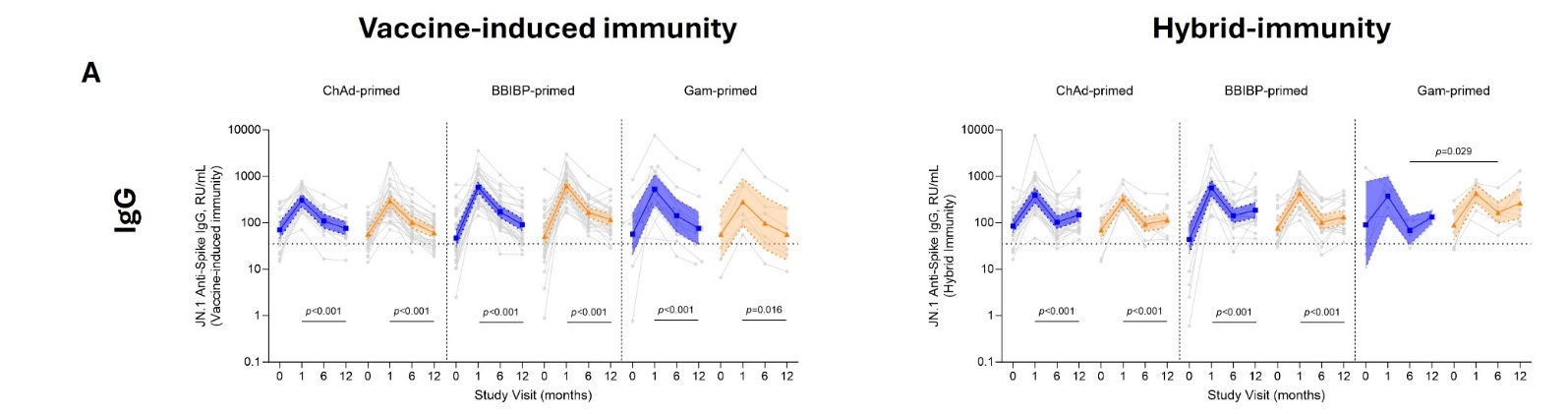

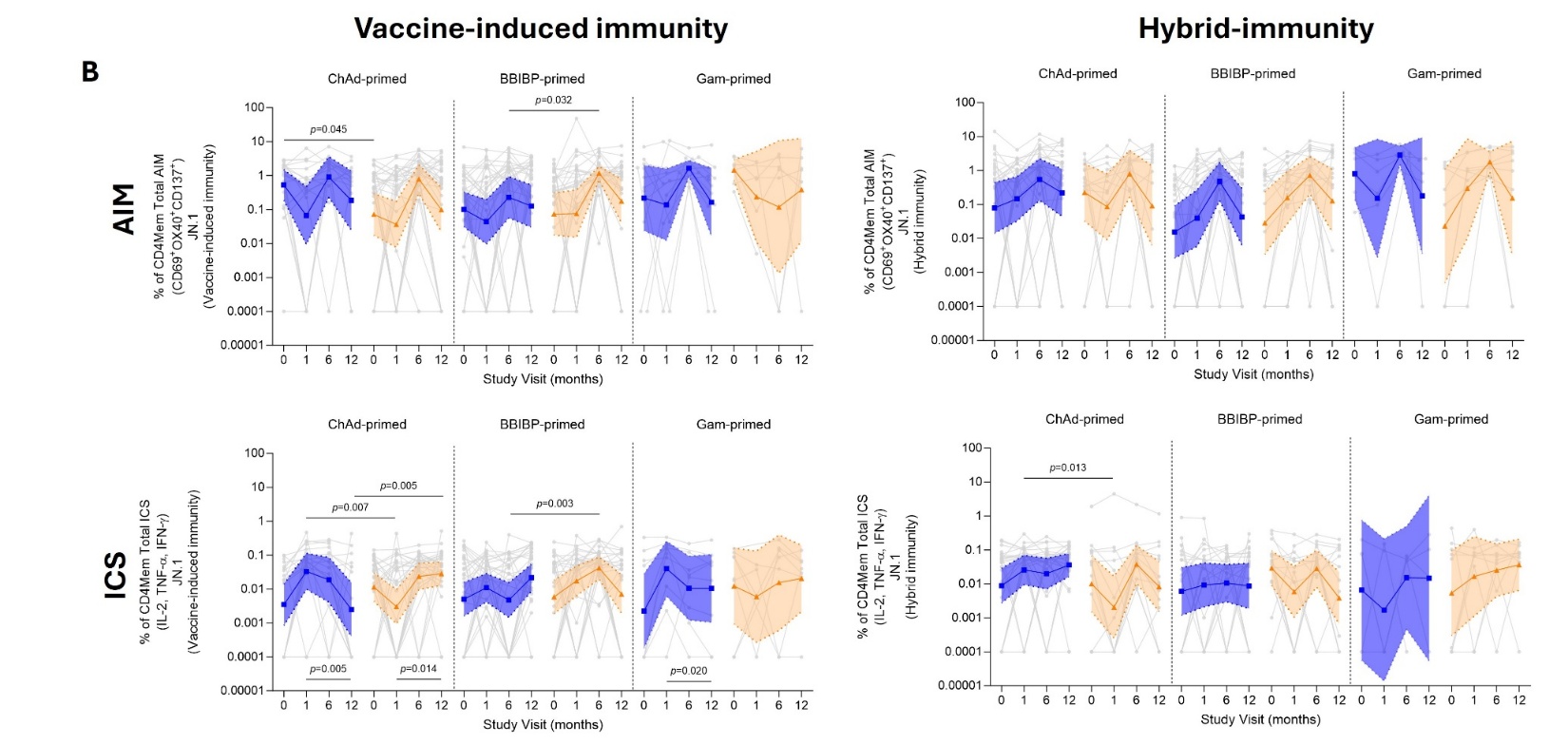


**Supplementary Figure 6*.* Vaccine-induced immunity (blue) and hybrid-immunity (pink) responses following fractional or standard dose booster vaccination against JN.1 variant.**  Vaccine-induced immunity and hybrid-immunity between 6-12 months post-vaccine was evaluated for (A) IgG and (B) CD4Mem AIM (CD60+, OX40+, CD137+) and ICS (IL-2+, TNF-α+, IFN-γ+) responses against JN.1 variant. Kinetics of these results are presented over the 12-month period. Responses over the 12-month period are presented as GMC ± 95% CI, with data log transformed for a parametric t-test performed between standard and fractional dose groups, with a paired Wilcoxon signed-rank test comparing day 28 and 12-months post vaccination.
